# Impact of short-term (24 h) blood pressure variability on 30-days clinical outcomes of acute strokes at two tertiary hospitals in Dar-es-Salaam

**DOI:** 10.3389/fstro.2025.1700321

**Published:** 2025-11-11

**Authors:** Ayubu Eliesikia Mashambo, Mandela Charles Makakala, Philip Babatunde Adebayo

**Affiliations:** 1 Department of Internal Medicine, Aga Khan University, Dar es Salaam, Tanzania; 2 The Aga Khan Hospital, Dar es Salaam, Tanzania

**Keywords:** blood pressure variability, modified Rankin Score, Barthel Index, acute stroke, Tanzania

## Abstract

**Background:**

Stroke remains the second leading cause of disability and death worldwide, with hypertension as its principal risk factor. Evidence from high-income countries indicates that blood pressure variability (BPV) is an independent predictor of outcomes during the acute phase, but data from African populations are limited despite the rising burden of cardiovascular disease. Understanding BPV patterns in this context is crucial for designing interventions to improve stroke outcomes.

**Objectives:**

To determine 24-h BPV patterns in acute stroke patients and assess their impact on 30-day functional outcomes.

**Methods:**

This multicenter prospective cohort study enrolled adults with acute stroke presenting within 72 h of symptom onset at two tertiary hospitals in Dar es Salaam. BPV patterns were measured using 24-h ambulatory monitoring, and demographic, clinical, and stroke subtype data were collected. Functional outcomes were evaluated using the modified Rankin Scale (mRS) and Barthel Index at admission, day 7, and day 30. Associations were examined using logistic regression.

**Results:**

Of 52 patients enrolled, 48 (92.3%) completed follow-up. Most were male (*n* = 31; 64.6%), middle-aged (47–60 years, *n* = 19; 39.6%), and hypertensive (*n* = 43; 91.5%). Hemorrhagic strokes (*n* = 27; 56.3%) demonstrated higher systolic and diastolic BP variability than ischemic strokes, though differences were not statistically significant. Impaired nocturnal dipping (day/night systolic BP ratio) was linked to poor outcomes in univariate analysis (*p* = 0.019) but lost significance in multivariate testing (*p* = 0.16). Functional outcomes improved significantly by day 30: the Barthel Index increased, and mRS scores decreased. NIHSS score at day 7 emerged as the strongest independent predictor of poor outcome (mRS: *p* = 0.027, OR = 3.04, 95% CI: 1.13–8.15). Higher education level was also associated with better functional outcomes (*p* = 0.03).

**Conclusion:**

In this Tanzanian cohort, reduced nocturnal dipping and elevated morning pressures were the most frequent BPV patterns, especially in hemorrhagic strokes, though not independently associated with outcomes at 30 days. Neurological severity at day 7 (NIHSS) was the strongest predictor of recovery, and lower education levels negatively influenced outcomes. The Barthel Index was more sensitive than the mRS in detecting functional gains.

## 1 Introduction

Stroke is defined as a neurological deficit resulting from acute focal injury to the central nervous system from vascular events such as cerebral infarction, intracerebral or subarachnoid hemorrhage (Sacco et al., 2013). It affects nearly 12 million people globally each year and is the second leading cause of death, responsible for over 7 million deaths and 160 million Disability Adjusted Life Years (DALYs) annually. Africa carries a disproportionate burden, with over 1.1 million new stroke cases annually, as well as more than 8 million people living with stroke, and young adults accounting for nearly 20% of stroke deaths. Since 1990, the incidence of stroke in Sub-Saharan Africa has increased by 85%, with the highest impact seen in rural areas and regions like East, West, and Central Africa, where DALYs and mortality continue to rise sharply (Feigin et al., 2025). Within this region, 30 day in hospital case fatality remains high: a 2024 pooled analysis reported that roughly one in four hospitalized patients with stroke die within 30 days, with hemorrhagic stroke, higher NIHSS, depressed GCS, and complications (e.g., aspiration, edema, fever, hyperglycemia) driving early mortality (Ackah et al., 2024).

A consistent contributor to poor outcomes in SSA is delayed arrival to definitive care, which limits access to reperfusion and organized stroke unit pathways. Multi-country and single-center studies show that the majority of patients reach hospital well beyond the thrombolysis/thrombectomy window. In Tanzania, the mean onset to arrival time for ischemic stroke was approximately 1.7 days, excluding patients with normal neuroimaging (Matuja et al., 2022). In South Africa, the median time to emergency department arrival was 33 h, with 81% arriving after 4.5 h and virtually none receiving thrombolysis (Khalema et al., 2018). Nigerian data similarly show that approximately 65% arrive more than 24 h after onset (Philip-Ephraim et al., 2015). Recent systematic reviews/meta-analyses across Africa confirm that only about 10% reach hospital within 3 h, whereas greater than 70% exceed standard treatment windows (Ganeti et al., 2025; Yakubu et al., 2025), with health system and socioeconomic barriers (low stroke literacy, transport costs, limited imaging/reperfusion capacity) frequently cited in Tanzanian settings (Michael et al., 2025). Such delays translate into missed opportunities for reperfusion, higher early mortality, and worse functional recovery trajectories.

In this context, blood pressure (BP) management remains a central, modifiable pillar of acute care both in the hyperacute window and among the many patients presenting after 24 h. Hypertension also plays a key role and contributes to blood pressure variability (BPV), a dynamic fluctuation in blood pressure that has emerged as a significant predictor of stroke outcomes independent of absolute blood pressure levels (Zhao et al., 2021; Zhang et al., 2018). With the burden of stroke continuing to grow in Africa, understanding local BPV patterns is essential for guiding interventions to improve outcomes and prevent complications.

The management of blood pressure in acute stroke is complex due to diverse etiologies and hemodynamic responses. While current guidelines recommend specific blood pressure targets in acute stroke care, the benefits of intensive blood pressure reduction remain inconclusive (Wajngarten and Silva, 2019). For acute ischemic stroke (AIS) not undergoing reperfusion, authoritative guidelines recommend permissive hypertension with treatment generally reserved for severely elevated BP (e.g., SBP > 220 mmHg or DBP > 120 mmHg); if treatment is initiated, a cautious reduction (15% over the first 24 to 48 h) is advised to avoid worsening penumbral hypoperfusion. In patients treated with intravenous thrombolysis/endovascular therapy, stricter ceilings (e.g., < 180/105 mmHg) are recommended to limit hemorrhagic transformation. After the initial 24 to 48 h, particularly in the late presenting majority in SSA, most guidance supports gradual blood pressure reduction toward secondary prevention targets as the patient stabilizes neurologically, with attention to comorbidities and autoregulatory status (Gorelick et al., 2020). For intracerebral hemorrhage (ICH), early intensive lowering (often targeting SBP to approximately 140 to 160 mmHg within the first hours) is recommended to reduce hematoma growth and improve outcomes, provided rapid lowering is feasible and safe. Beyond the hyperacute phase, continued blood pressure control is essential to prevent rebleeding, edema progression, and later events, with individualized targets informed by the 2022 AHA/ASA ICH guideline and local resource constraints (Greenberg et al., 2022). Beyond absolute blood pressure levels, the role of BPV as an independent predictor of stroke prognosis cannot be overstated. Importantly, BPV can occur across various timeframes, including very short-term (over seconds or minutes), short-term (e.g., 24-h), and day-to-day variability, with circadian patterns such as early morning surges, day/night ratio and nocturnal dipping percentage shown to influence prognosis (Parati et al., 2018).

Notably, elevated short term and long term BPV correlates with target organ damage and worse functional outcomes post-stroke influenced by intrinsic factors such as autonomic function, stroke severity, and psychological stress (Parati, 2005). Additionally, meta-analyses and multicenter trials have supported the association between increased BPV and poor stroke outcomes, including a study by Manning et al. (2015) that demonstrated that increased systolic short-term BPV measured within 48 to 72 h of admission is linked to unfavorable modified Rankin Scale (mRS) scores with pooled odds ratios indicating significantly elevated risk of disability. Also, large-scale trials, including multicenter studies like the one by Minhas et al. (2019) have further demonstrated that BPV is associated with 90-day poor outcomes after stroke, regardless of stroke subtype or intervention such as head positioning. Similarly, *post hoc* studies in China and the USA found strong associations between elevated BPV especially during the early morning and poor short- and long-term outcomes, including mortality and severe disability. While some studies, especially in the UK (Manning et al., 2015), reported no significant association between BPV and short-term outcomes, the overall body of evidence supports BPV as a meaningful prognostic marker.

Importantly, ethnic differences in BPV have been observed, with African Americans displaying higher BPV and attenuated nocturnal BP declines compared to European Americans. These disparities may arise from differences in stress reactivity, vasoactive mechanisms, and sodium handling. Such patterns may contribute to the disproportionate burden of hypertension and stroke-related complications in Black populations (Harshfield et al., 2002). In Black populations, unique features such as low-renin hypertension partly driven by genetic polymorphisms like the T594M mutation of the epithelial sodium channel may contribute to higher BPV and a greater burden of hypertension-related complications (Williams et al., 2014; Li et al., 2010). The latter necessitating research within this specific population to inform the development of targeted interventions.

Despite this, the relevance of BPV in African stroke populations remains underexplored. Most evidence comes from high-income countries, limiting generalizability to African populations with distinct hypertension profiles and healthcare challenges. Additionally, the impact of early BPV patterns on outcomes in African stroke patients remain unclear. Only recently, a study in Uganda (Kulaba et al., 2023) highlighted that systolic BPV was independently associated with early mortality in both ischemic and hemorrhagic stroke, marking the first such evidence from Sub-Saharan Africa, with a need for further studies to confirm and contextualize these findings. Consequently, this study aimed to investigate BPV during the first 24 h of acute stroke and its association with functional outcomes in tertiary hospitals in Dar-es-Salaam, Tanzania, addressing a significant gap in the African stroke research landscape.

## 2 Methods

### 2.1 Research design

This multicenter prospective cohort study was conducted at two tertiary care institutions in Tanzania: Aga Khan Hospital, Dar es Salaam, and Muhimbili National Hospital (MNH) Mloganzila. Both hospitals house internal medicine departments with general wards, high dependency units (HDU), and intensive care units (ICU), along with 24-h emergency and radiology services capable of performing computed tomography (CT) and magnetic resonance imaging (MRI) scans.

The study population comprised all acute stroke patients admitted to the two tertiary hospitals between September 2024 and December 2024. Those enrolled into the study were adult patients (≥18 years) presenting with acute stroke confirmed by imaging (CT/MRI) within 72 h of symptom onset. Persons with a history of prior stroke, poor pre-admission functional status (mRS >5), or a Glasgow Coma Scale (GCS) < 8 were excluded from the study as improvement of function is not be easily identified in the presence of residual deficit or poor level of consciousness.

Participants were consecutively sampled as they presented to the emergency department. The sample size was determined using G^*^Power (Faul et al., 2007), based on a medium effect size (f^2^ = 0.15), 80% power, 95% confidence level, and one predictor variable in a linear regression model, resulting in a minimum of 81 participants. Allowing for a 10% attrition rate and drawing on recent epidemiological evidence from Tanzania and sub-Saharan Africa where stroke incidence is reported at 316 per 100,000, prevalence reaches 1,460 per 100,000, and 3-year fatality rates exceed 80% (Ngimbwa et al., 2025), the final adjusted sample size was determined to be 89 participants.

### 2.2 Study variables

The primary outcome was functional status measured using the modified Rankin Scale (mRS) and the Barthel Index. The main independent variable was 24-h blood pressure variability (BPV), calculated using standard deviation and coefficient of variation of systolic and diastolic blood pressure (BP). Additional covariates included demographic factors (age, sex, body mass index, marital status), clinical characteristics (stroke type, location, severity via National Institute of Health Stroke Scale), comorbidities (diabetes, hypertension, coronary heart disease), behavioral factors (smoking, alcohol use), lab values (lipid profile, glucose, troponin), imaging features (stroke volume, cerebral edema, midline shift), treatment factors (antihypertensive/reperfusion therapy, head position, hyperthermia on admission) and time from LKW (last known well) to hospital arrival or onset of BPV measurement.

### 2.3 Data collection and materials

BP measurements were taken using an ABPM-50 CONTEC ambulatory BP monitor (Contec Medical Systems Co. Ltd., 2018) attached to the non-dominant arm. BP readings were recorded every 15 min during the day and every 30 min at night for the first 24-h post admission. BPV patterns such as dipping status, nocturnal dipping percentage, and morning blood pressures (MBPS) were derived using ABPM software (Contec Medical Systems Co. Ltd., 2018). MBPS was classified based on the difference in mean BP values 2 h before and after wake-up.

**Figure 1:**
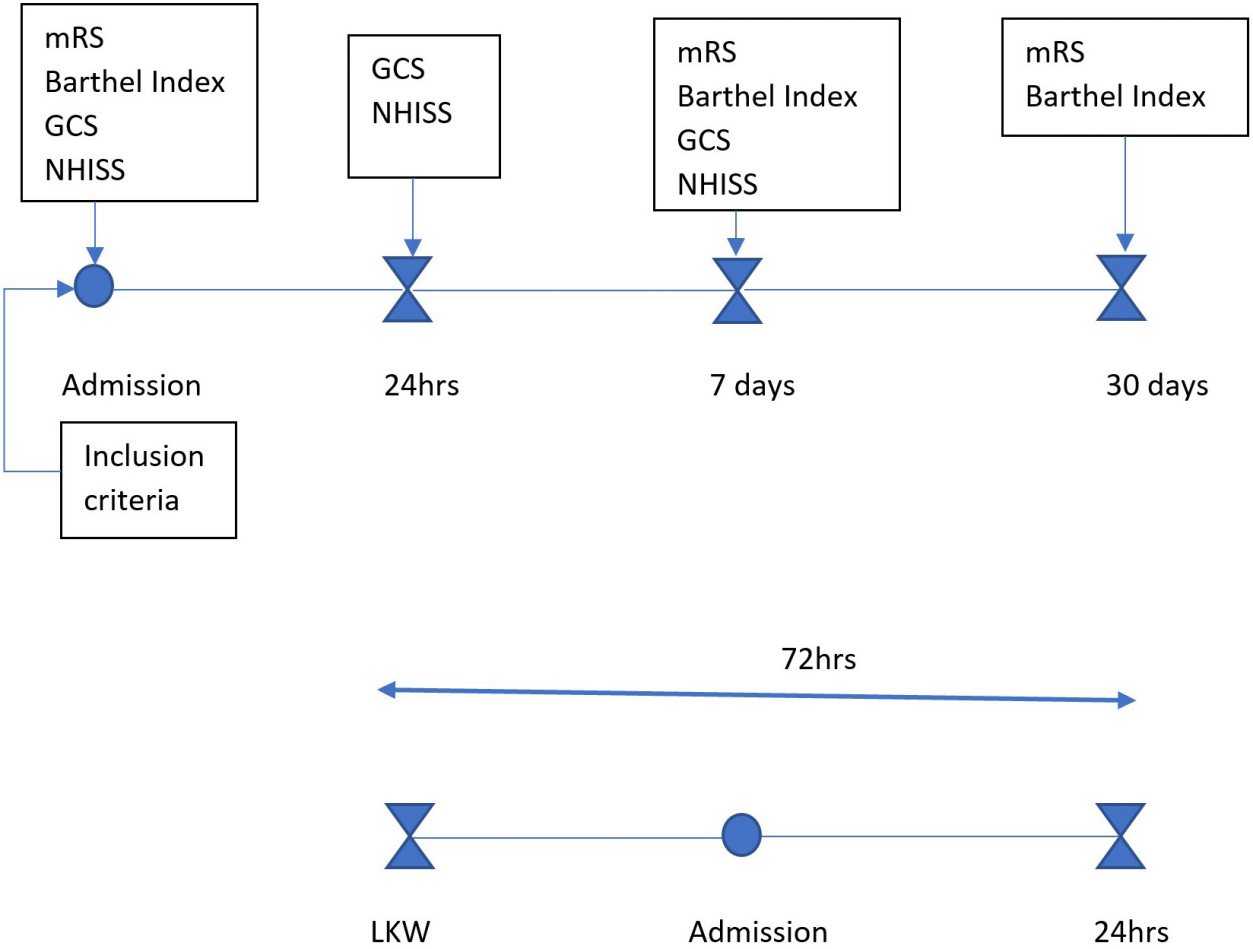
Data collection flow chart: patients who presented within 72 h from last known well were assessed after meeting the inclusion criteria. Baseline assessments (mRS, Barthel Index, GCS, NIHSS) were conducted at admission, followed by neurological reassessment at 24 h (GCS, NIHSS), at day 7 (mRS, Barthel Index, GCS, NIHSS), and at day 30 (mRS, Barthel Index) to determine functional outcomes.

Patients were assessed by the principal investigator (PI) and co-investigators using NIHSS and GCS at baseline, 24 h, and day 7. Functional status was assessed with mRS and Barthel Index (Appendices 1, 4) at admission, day 7, and 30 days via phone follow-up when necessary, using revised simplified mRS (Appendices 2, 3). Data collection was performed using paper-based questionnaires and included socio-demographic, clinical, and behavioral information obtained from patient files and family members. Questionnaires were interviewer-administered by members of the study team and trained research assistants. Anthropometric data were collected as per World Health Organization (WHO) guidelines. The data collection flow chart below (Figure 1) elaborates the process of data collection to follow up.

### 2.4 Data analysis

Data were digitized, cleaned, and managed securely in a password-protected excel sheet accessible only to the PI. Statistical analysis was conducted using Jamovi version 2.6.13 (Love et al., 2025). Descriptive statistics (counts and proportions, means and standard deviation, or median and interquartile range based on data distribution) summarized baseline data. Categorical variables were compared using Pearson's chi-square test; continuous variables were tested for normality using the Shapiro-Wilk test. Independent *t*-tests were used to explore associations between BPV and functional outcomes. Those variables with *p* < 0.25 were moved to multivariable logistic regression and linear regression models which assessed the relationship between BPV and functional status (mRS, Barthel), with results presented as odds ratios (OR) and 95% confidence intervals. A *p*-value < 0.05 was considered statistically significant.

### 2.5 Ethical consideration

Ethical approval was obtained from AKU and MNH-Mloganzila ethics committees (Ref: AKU/2024/01/fb/03/113 and JA.294/428/01C/021 respectively). Written informed consent was obtained from all participants or their caregivers. Confidentiality was ensured using unique study identifiers.

## 3 Results

### 3.1 Descriptive statistics

**Figure 2:**
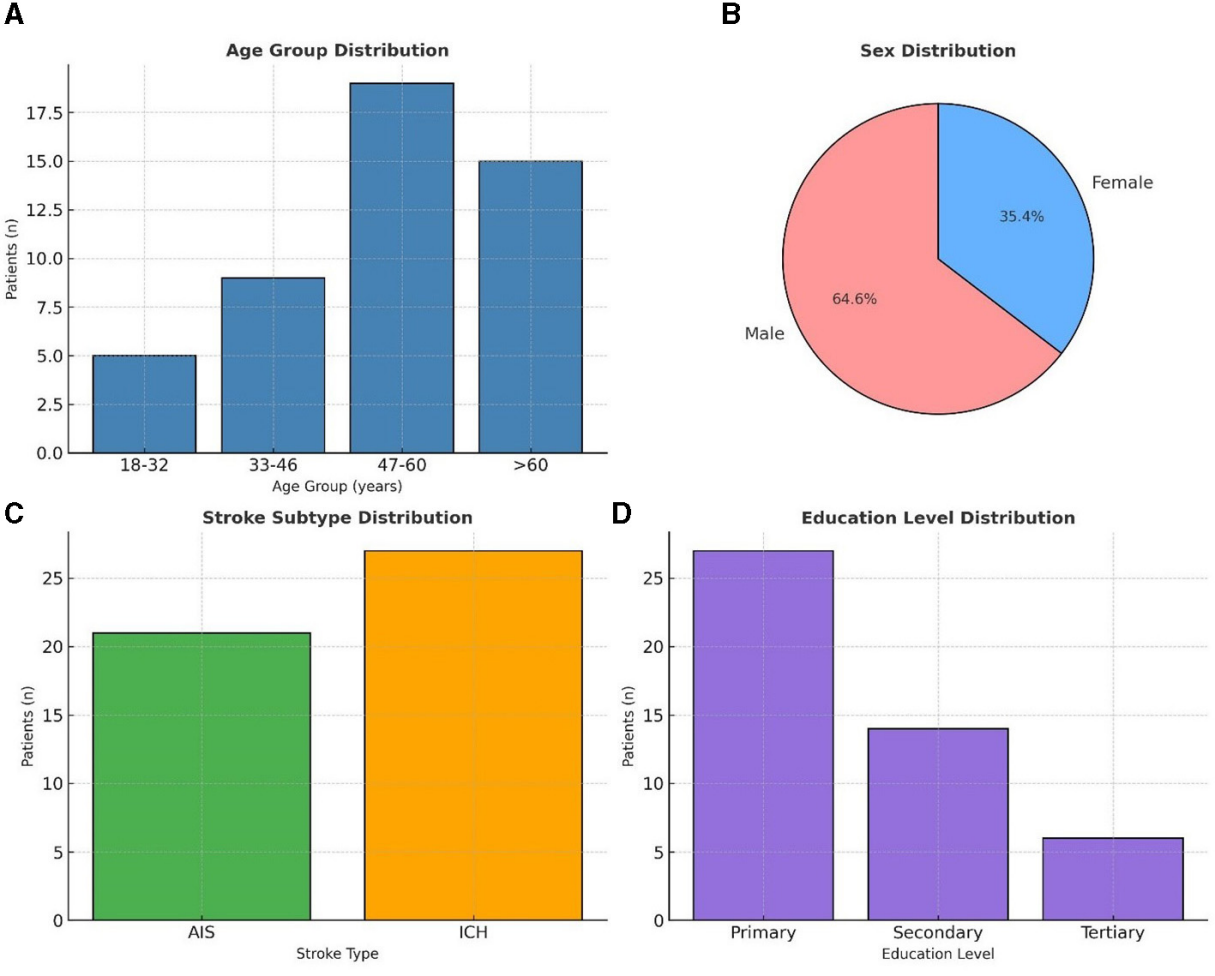
Demographic characteristics (n = 48) **(A)** shows the age group distribution of patients, with the majority between 47–60 years. **(B)** illustrates the sex distribution, with males comprising 64.6% and females 35.4%. **(C)** presents stroke subtype distribution, with intracerebral hemorrhage (ICH) more frequent than acute ischemic stroke (AIS). **(D)** depicts education level distribution, with most patients having primary education.

We set out to recruit a total of 89 patients but enrolled 52 patients (58.4 % response rate). Of the latter, only 48 patients completed the study, reflecting a modest attrition rate of 7.7%. The cohort was predominantly male (*n* = 31; 64.6%) and middle-aged, (47–60 years, *n* = 19; 39.6%), with most participants residing in urban Dar-es-Salaam and having a primary-level education (*n* = 27; 57.4%) (Figure 2). Hypertension emerged as the most common comorbidity, affecting over 90% (*n* = 43). Similarly behavioral risk factors such as alcohol consumption and recent tobacco smoking were each reported in over a quarter of the sample (*n* = 13, 27.1%, respectively). Despite the presence of these known vascular risk factors, no participants were found to have chronic kidney disease or atrial fibrillation (Supplementary Table 1).

**Figure 3:**
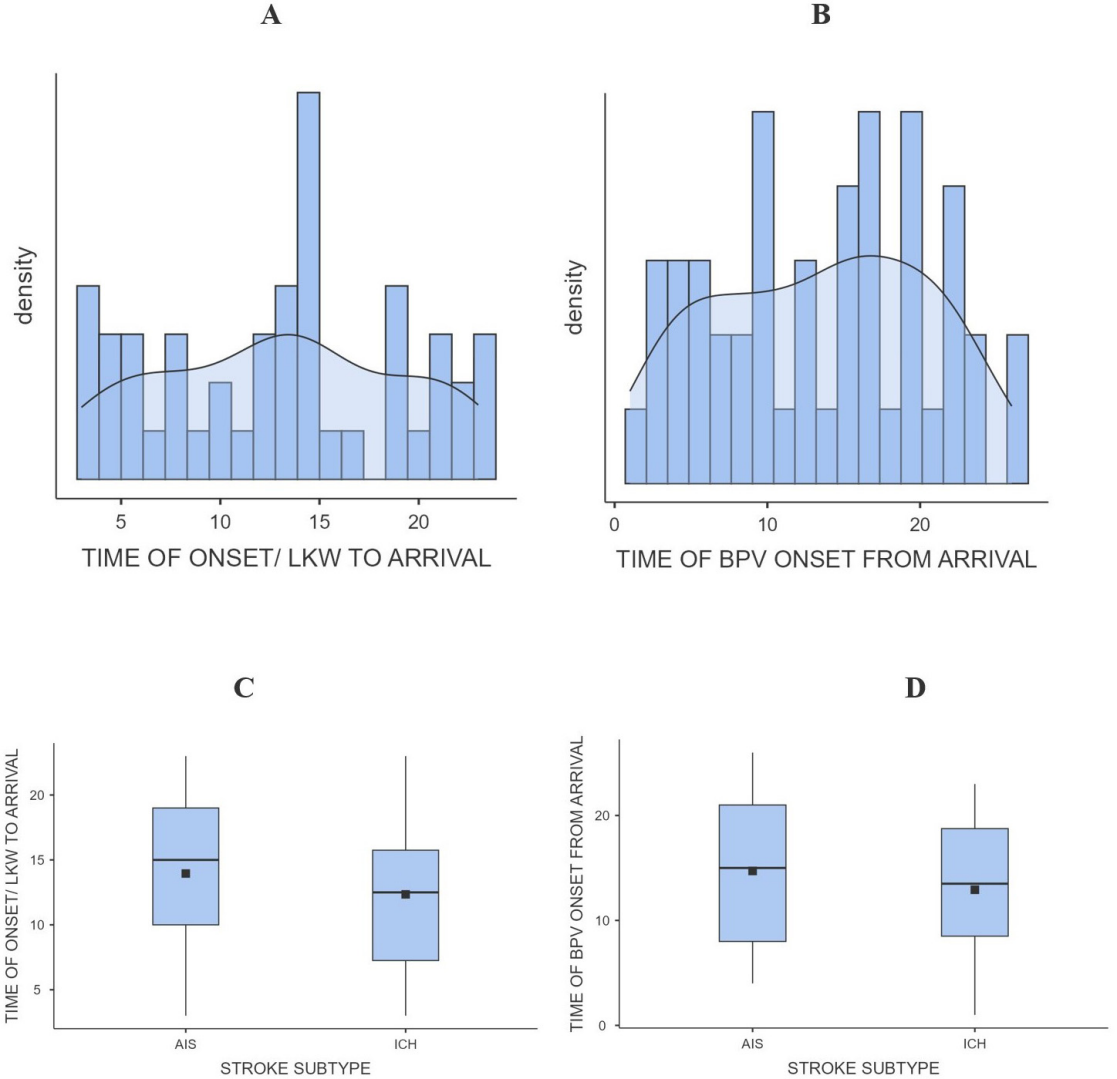
Bar/density graph **(A)** and **(B)**, box and whisker graph **(C)** and **(D)** for time of arrival and BPV onset from LKW.

The average time from last known well (LKW) to hospital arrival differed between ischemic and hemorrhagic stroke patients. Patients with acute ischemic stroke (AIS) tended to arrive later compared to those with intracerebral hemorrhage (ICH). Time of arrival was stratified to > 24 h and < 24 h. For AIS, the mean arrival time was 10.6 h in patients who presented within 24 h, and 15.3 h among those arriving after 24 h. In comparison, ICH patients had slightly shorter times, averaging 9.5 h in the < 24-h group and 14.4 h in the ≥24-h group. Overall, the median arrival time was approximately 15 h for AIS and 13 h for ICH, reflecting modest but consistent delay in arrival in ischemic stroke (Figure 3C). Nearly half of the cohort (45.8%) presented within 24 h of symptom onset, while the remainder arrived later.

After arrival, patients were initiated on blood pressure variability (BPV) monitoring with average time from arrival to BPV onset ranging from 8.0 to 17.1 h in AIS patients, and 8.1 to 16.5 h in ICH patients. This pattern suggests broadly similar in-hospital initiation times between the two stroke subtypes, although AIS patients tended to begin BPV monitoring slightly later. Figure 3D, demonstrates this distribution, with overlapping ranges for both groups. When combining these intervals, the total time from LKW to BPV onset was longest among AIS patients. For those arriving within 24 h, BPV monitoring began after a mean of 18.6 h in AIS vs. 17.6 h in ICH. Among patients presenting beyond 24 h, BPV initiation occurred after 32.4 h in AIS compared to 30.9 h in ICH. These findings indicate that AIS patients experienced a modest but consistent delay in both arrival and initiation of BPV monitoring compared to ICH patients across all time categories (Table 1 and Figure 3).

**Table 1:** Time of arrival, LKW, and BPV onset, with stroke subtypes.

**Stroke subtype**	**Time group**	***N* (%)**	**Mean arrival from LKW (h)**	**Mean BPV onset from arrival (h)**	**Mean total LKW → BPV (h)**
AIS	< 24 h	7 (14.6%)	10.6	8.0	18.6
AIS	≥24 h	15 (31.2%)	15.3	17.1	32.4
ICH	< 24 h	11 (22.9%)	9.5	8.1	17.6
ICH	≥24 h	15 (31.2%)	14.4	16.5	30.9

**Figure 4:**
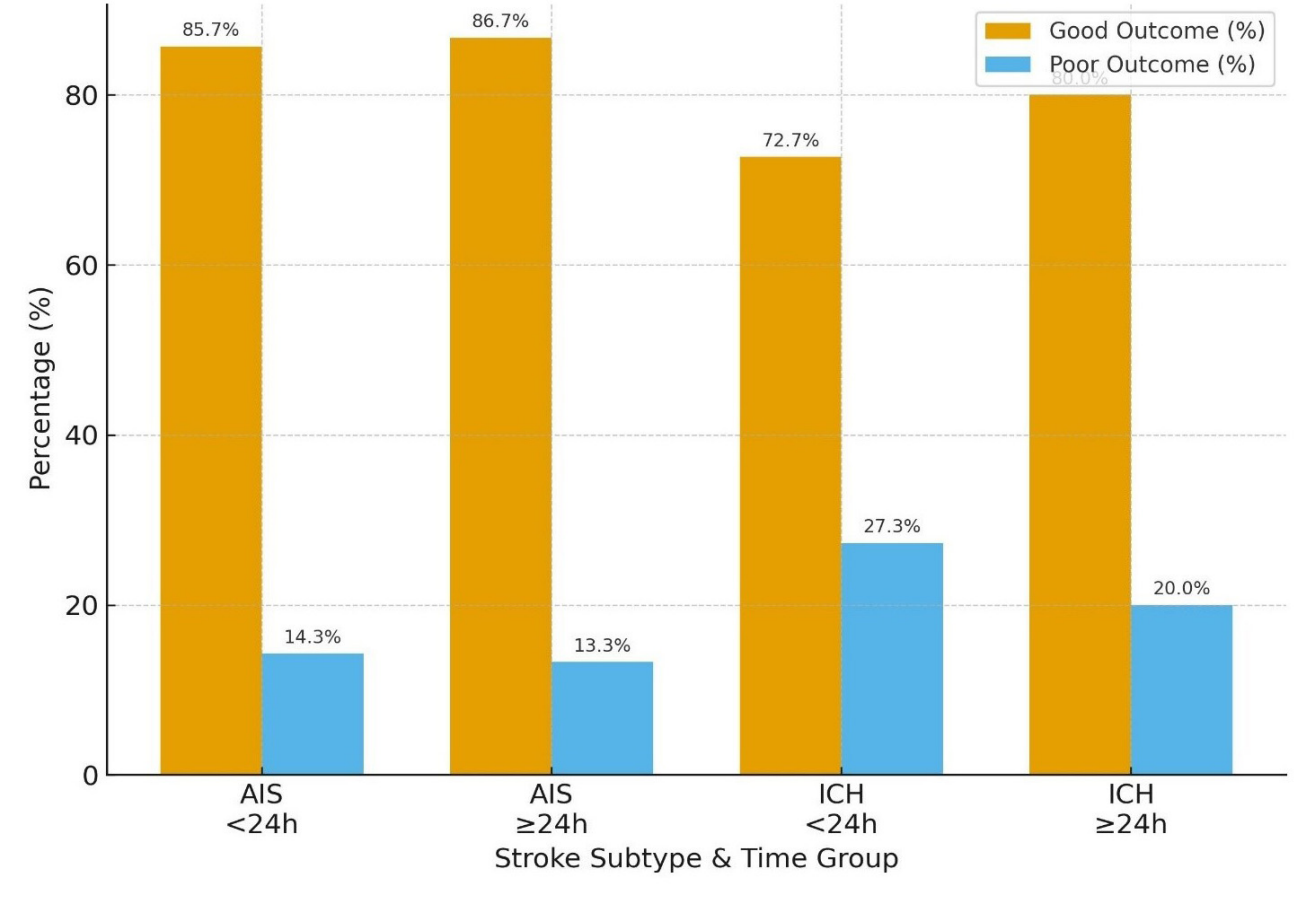
mRS score (good or poor) in AIS vs. ICH in those patients arriving < 24 h and >24 h from LKW.

When functional outcomes at 30 days were stratified by stroke subtype and time to BPV onset (< 24 h vs. ≥24 h from LKW), distinct patterns emerged. For the modified Rankin Scale (mRS), the majority of AIS patients achieved good outcomes irrespective of timing: 85.7% in the < 24 h group and 86.7% in the ≥24 h group. Among ICH patients, good outcomes were slightly less frequent, observed in 72.7% of those arriving < 24 h and 80.0% in those ≥24 h. Poor outcomes were more common in ICH compared to AIS, particularly in the early (< 24 h) group (27.3% vs. 14.3%) (Table 2 and Figure 4).

**Table 2:** Modified Rankin Scale (mRS) outcomes at 30 days by stroke subtype and time from last known well to BPV onset (< 24 h vs. ≥24 h).

**Stroke subtype**	**Time group**	**mRS at 30 days**	
		**Good** ***N*** **(%)**	**Poor** ***N*** **(%)**
**AIS**	< 24 h	6 (85.7%)	1 (14.3%)
	≥24 h	13 (86.7%)	2 (13.3%)
**ICH**	< 24 h	8 (72.7%)	3 (27.3%)
	≥24 h	12 (80.0%)	3 (20.0%)

mRS, modified Rankin score; AIS, Acute Ischaemic Stroke; ICH, Intracerebral hemorrhage.

**Figure 5:**
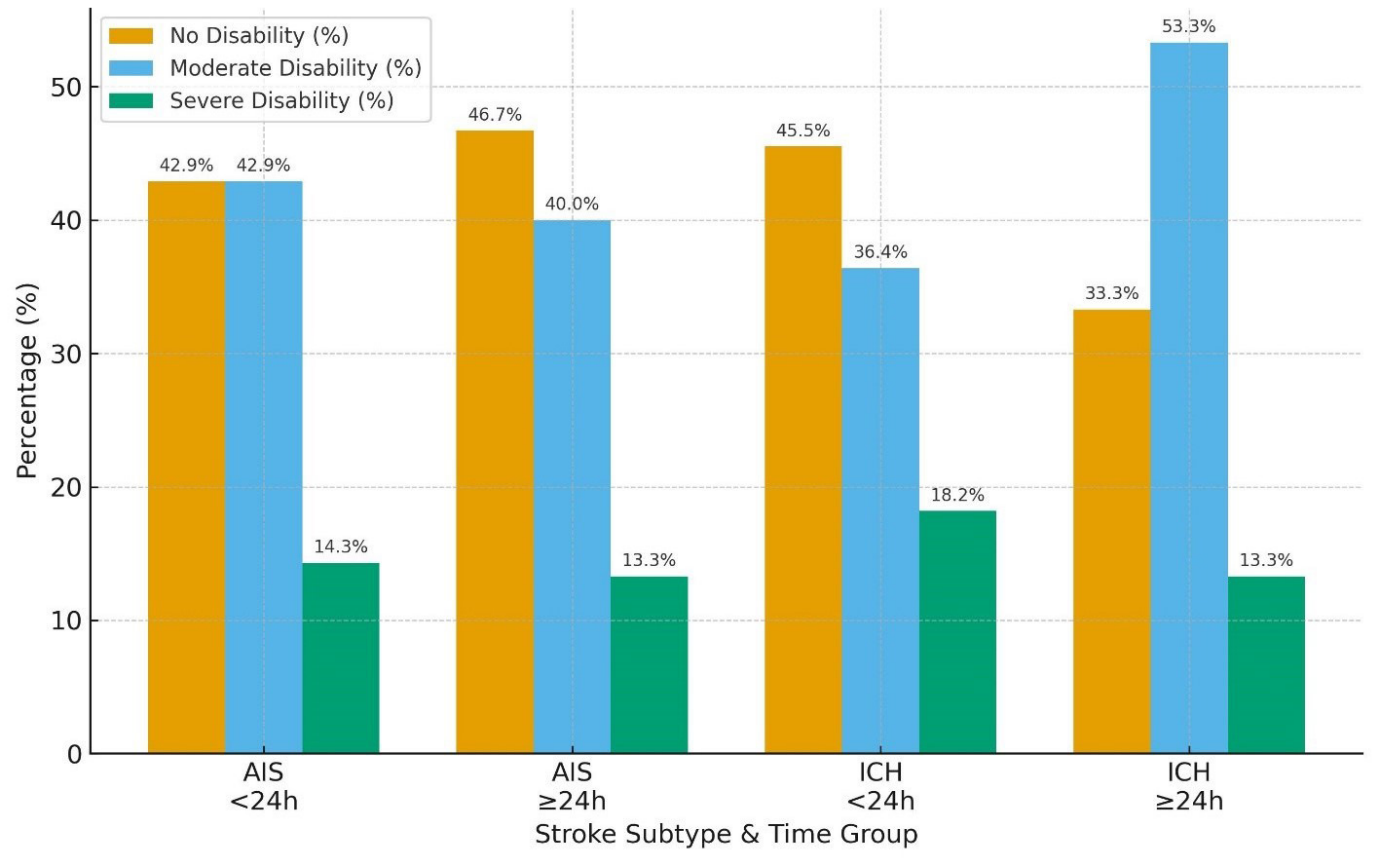
Barthel Index graph for AIS vs. ICH in patients arriving < 24 h vs. >24 h from LKW.

Regarding the Barthel Index, functional independence was also more favorable in AIS than ICH. In the AIS cohort, nearly equal proportions of patients in the < 24 h group achieved no disability (42.9%) or moderate disability (42.9%), with only 14.3% experiencing severe disability. Similar distributions were seen in the ≥24 h group (no disability 46.7%, moderate 40.0%, severe 13.3%). In contrast, ICH patients demonstrated a higher burden of functional impairment: among those < 24 h, 45.5% attained no disability while 36.4% had moderate and 18.2% severe disability; in the ≥24 h group, moderate disability predominated (53.3%), with fewer achieving no disability (33.3%) and 13.3% remaining severely disabled (Table 3 and Figure 5).

**Table 3:** Barthel Index outcomes at 30 days by stroke subtype and time from last known well to BPV onset (< 24 h vs. ≥24 h).

**Stroke subtype**	**Time group**	**Barthel Index**		
		**No disability** ***N*** **(%)**	**Moderate disability** ***N*** **(%)**	**Severe disability** ***N*** **(%)**
**AIS**	< 24 h	3 (42.9%)	3 (42.9%)	1 (14.3%)
	≥24 h	7 (46.7%)	6 (40.0%)	2 (13.3%)
**ICH**	< 24 h	5 (45.5%)	4 (36.4%)	2 (18.2%)
	≥24 h	5 (33.3%)	8 (53.3%)	2 (13.3%)

AIS, Acute Ischaemic Stroke; ICH, Intracerebral hemorrhage.

**Figure 6:**
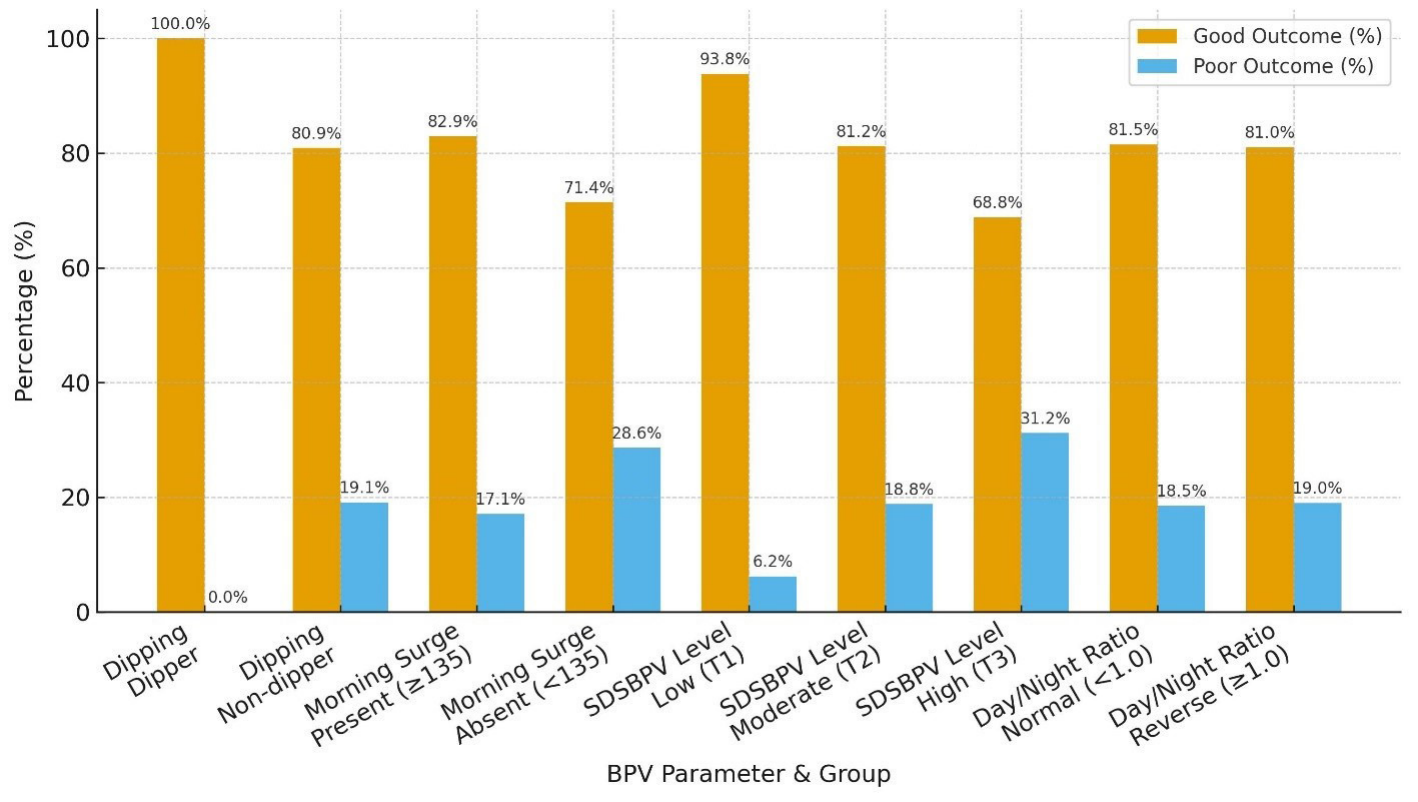
mRS outcomes (poor vs. good) in different BPV parameters.

Functional outcomes at 30 days varied across BPV parameters. Non-dippers had a higher proportion of poor outcomes compared to dippers, while morning surge was associated with a greater share of moderate disability on the Barthel Index despite most patients achieving good mRS. Higher SDSBPV (third tercile**)** correlated with worse functional status, with 31.2% experiencing poor mRS and nearly one in five having severe disability. By contrast, patients in the low variability tercile (first tercile) showed the most favorable outcomes. The day/night ratio (normal vs. reverse dipper) showed only minor differences, with broadly similar mRS outcomes, though reverse dippers tended to have slightly fewer severe disability cases on the Barthel Index (Tables 4, 5 and Figure 6).

**Table 4:** mRS at 30 days by BPV parameters.

**BPV parameter**	**Group**	**mRS at 30 days**	
		**Good** ***N*** **(%)**	**Poor** ***N*** **(%)**
Dipping	Dipper	1 (100.0%)	0 (0.0%)
	Non-dipper	38 (80.9%)	9 (19.1%)
Morning surge	Present (≥135)	34 (82.9%)	7 (17.1%)
	Absent (< 135)	5 (71.4%)	2 (28.6%)
SDSBPV level	(T1) low variability	15 (93.8%)	1 (6.2%)
	(T2) moderate	13 (81.2%)	3 (18.8%)
	(T3) high	11 (68.8%)	5 (31.2%)
Day/night ratio	Normal (< 1.0)	22 (81.5%)	5 (18.5%)
	Reverse dipper (≥1.0)	17 (81.0%)	4 (19.0%)

BPV, Blood pressure variability; mRS, modified Rankin Score; T1, first tercile; T2, second tercile; T3, third tercile.

**Table 5:** Barthel Index at 30 days by BPV parameters.

**BPV parameter**	**Group**	**Barthel Index at 30 days**		
		**No disability** ***N*** **(%)**	**Moderate disability** ***N*** **(%)**	**Severe disability** ***N*** **(%)**
Dipping	Dipper	1 (100.0%)	0 (0.0%)	0 (0.0%)
	Non-dipper	19 (40.4%)	21 (44.7%)	7 (14.9%)
Morning surge	Present (≥135)	16 (39.0%)	20 (48.8%)	5 (12.2%)
	Absent (< 135)	4 (57.1%)	1 (14.3%)	2 (28.6%)
SDSBPV level	(T1) low variability	5 (31.2%)	10 (62.5%)	1 (6.2%)
	(T2) moderate	9 (56.2%)	4 (25.0%)	3 (18.8%)
	(T3) high	6 (37.5%)	7 (43.8%)	3 (18.8%)
Day/night ratio	Normal (< 1.0)	10 (37.0%)	12 (44.4%)	5 (18.5%)
	Reverse dipper (≥1.0)	10 (47.6%)	9 (42.9%)	2 (9.5%)

BPV, Blood pressure variability; T1, first tercile; T2, second tercile; T3, third tercile.

**Figure 7:**
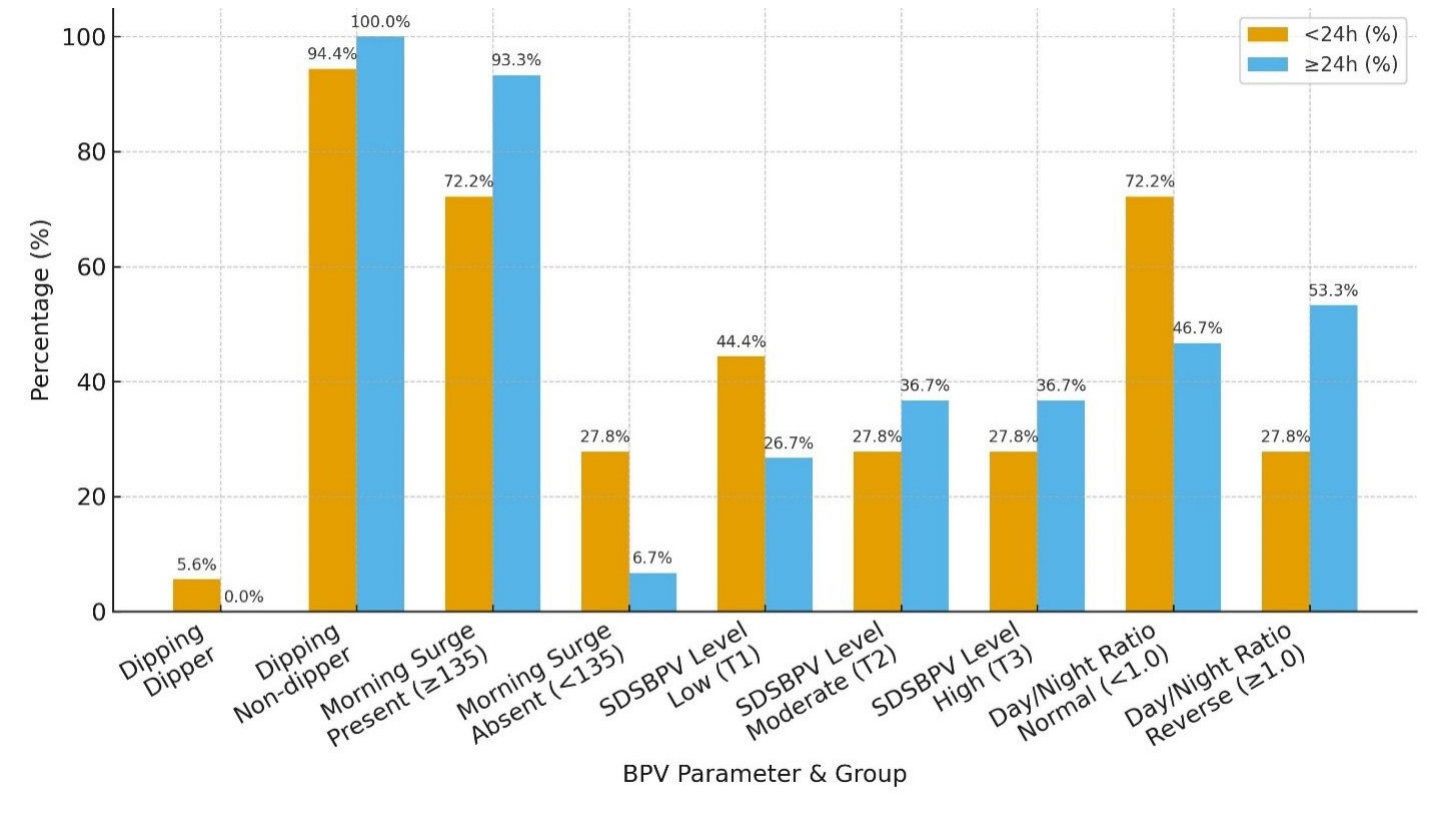
BPV parameters of patients arriving < 24 h vs. >24 h.

When patients were stratified by time from last known well to BPV onset, distinct patterns in BPV parameters were observed. Nearly all patients in both groups were non-dippers, with only a single dipper identified in the < 24 h group. Morning surge was more frequent among those initiated at ≥24 h (93.3%) compared with the < 24 h group (72.2%). Distribution across SDSBPV terciles suggested that early (< 24 h) monitoring captured more patients with low variability (44.4%), whereas later (≥24 h) monitoring showed a higher proportion in the moderate and high variability categories (36.7% each). Regarding day/night ratio, reverse dipping was more common in the ≥24 h group (53.3%) compared to the < 24 h group (27.8%), while a normal circadian pattern predominated in those monitored earlier (Table 6 and Figure 7).

**Table 6:** Time from LKW to BPV onset vs. BPV parameters.

**BPV parameter**	**Group**	** < 24 h *N* (%)**	**≥24 h *N* (%)**
Dipping	Dipper	1 (5.6%)	0 (0.0%)
	Non-dipper	17 (94.4%)	30 (100.0%)
Morning surge	Present (≥135)	13 (72.2%)	28 (93.3%)
	Absent (< 135)	5 (27.8%)	2 (6.7%)
SDSBPV level	(T1) low variability	8 (44.4%)	8 (26.7%)
	(T2) moderate	5 (27.8%)	11 (36.7%)
	(T3) high	5 (27.8%)	11 (36.7%)
Day/night ratio	Normal (< 1.0)	13 (72.2%)	14 (46.7%)
	Reverse dipper (≥1.0)	5 (27.8%)	16 (53.3%)

SDSBPV, Standard Deviation Systolic Blood Pressure Variability; BPV, Blood pressure Variability; LKW, Last Known Well.

Laboratory evaluations, however revealed moderate variability in renal function, as reflected by serum creatinine, while hemoglobin levels were relatively stable across participants. Whilst the values of hemoglobin were largely within range, the serum creatinine showed some modest elevations across some participants (Table 7).

**Table 7:** Laboratory variables.

**Variable**	**Median (IQR)**	**Minimum**	**Maximum**
RBG	6.70 (1.90)	4.10	14.9
Creatinine	111.00 (63.30)	61.00	178.0
Hemoglobin	14.00 (3.00)	10.00	16.9

Imaging studies predominantly involved CT scans (*n* = 41, 85.4%), with a minority undergoing both CT and MRI. Intracerebral hemorrhage (ICH) (*n* = 27, 56.3%) was more common than ischemic stroke (AIS) in this cohort. Among those with AIS, large artery atherosclerosis (*n* = 16, 76.2%) was the most frequently observed subtype. Notably, no cases of cardioembolic stroke were identified (Table 8).

**Table 8:** Stroke variables.

**Variable**	**Category**	**Frequency (%)**
Brain imaging	CT head	41 (85.4)
	Both	7 (14.6)
Stroke type	AIS	21 (43.8)
	ICH	27(56.3)
TOAST classification	Small vessel occlusion	5 (23.8)
	Large artery atherosclerosis	16 (76.2)
	Cardioembolic	0
Hemorrhagic subtype	Intracerebral	26 (96.3)
	Subarachnoid	1
Head position	Flat	4
	Upright	44 (91.7)
Reperfusion therapy	Yes	4
	No	44 (91.7)

**Figure 8:**
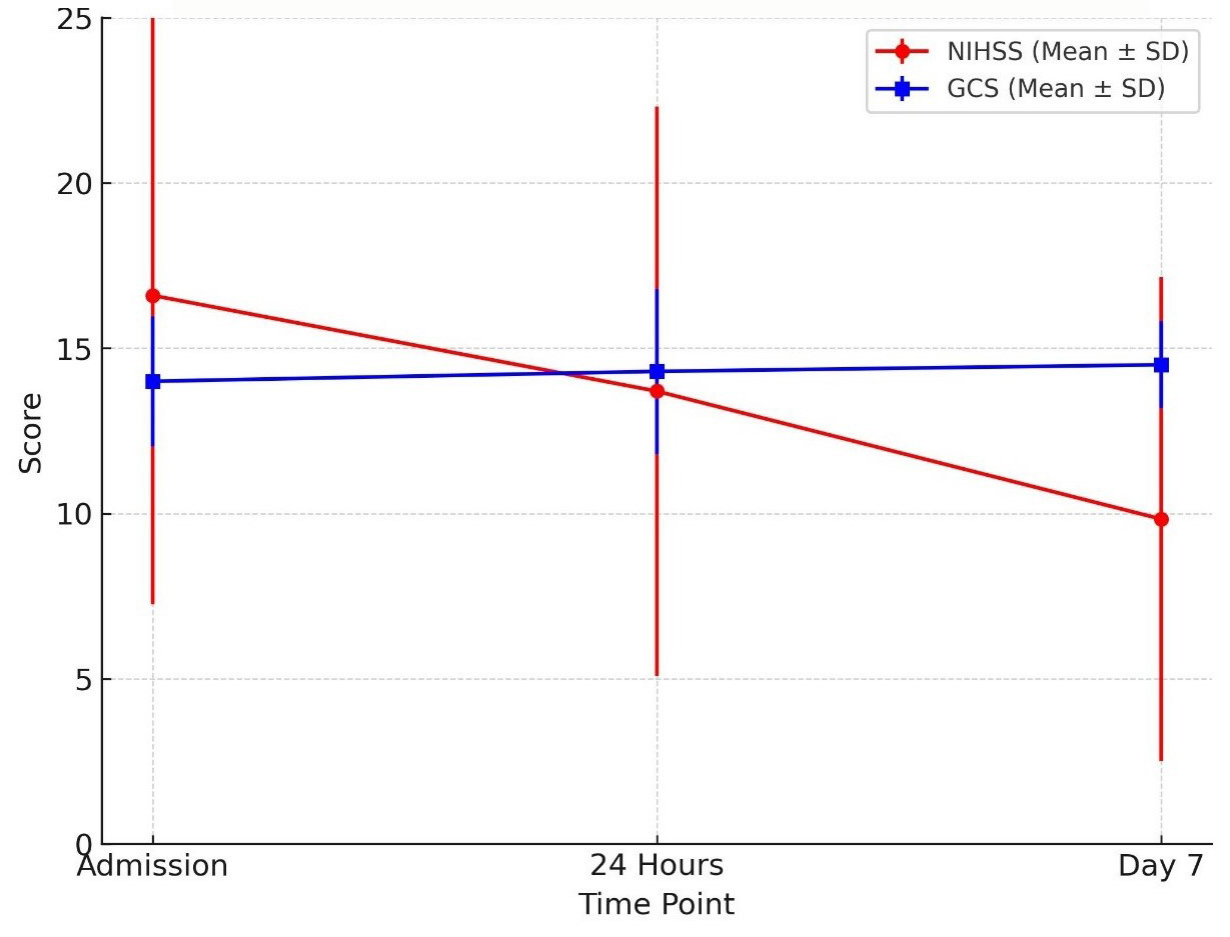
Neurological recovery over time trends in neurological status during the first week of admission. The mean NIHSS score declined from admission to day 7, indicating clinical improvement, while mean GCS scores showed a slight upward trend. Error bars represent standard deviations.

The NIHSS scores declined over the observation period, with the most notable changes observed by day 7. Similarly, GCS scores remained largely unchanged, with consistently high mean values (14.0 to 14.5) throughout the period (Table 9 and Figure 8). However, statistical testing using the Friedman test did not reveal significant differences in NIHSS or GCS across time points; NIHSS (χ^2^**:** 2.47, *p* = 0.291) and GCS (χ^2^**:** 4.07, *p* = 0.131) (Table 10).

**Table 9:** NIHSS score and GCS variables.

**Score type**	**Time point**	**Mean (SD)**	**Median (IQR)**
NIHSS score	At admission	16.6 (9.34)	17 (13.3)
	At 24 h	13.7 (8.62)	13 (14.0)
	At 7 days	9.83 (7.33)	10 (11.3)
GCS	At admission	14.0 (1.97)	15 (1)
	At 24 h	14.3 (2.50)	15 (1)
	At 7 days	14.5 (1.32)	15 (0)

NIHSS, National Institute of Health Stroke Scale; GCS, Glasgow Coma Scale.

**Table 10:** Friedman test summary table for NIHSS and GCS scores across the three time points.

**Score type**	**Test statistic (χ^2^)**	***p*-value**
NIHSS	2.47	0.291
GCS	4.07	0.131

NIHSS, National Institute of Health Stroke Scale; GCS, Glasgow Coma Scale.

Importantly, the inverse correlation between NIHSS and GCS scores at all time points was strong and statistically significant [at admission (r: −0.76, *p* < 0.001), 24 h (r: −0.72, *p* < 0.001), day 7 (r: −0.68, *p* < 0.001)] (Table 11).

**Table 11:** Correlation between NIHSS and GCS at each time point.

**Time point**	**Pearson correlation (r)**	***p*-value**
Admission	−0.76	< 0.001
24 h	−0.72	< 0.001
Day 7	−0.68	< 0.001

NIHSS, National Institute of Health Stroke Scale; GCS, Glasgow Coma Scale.

**Figure 9:**
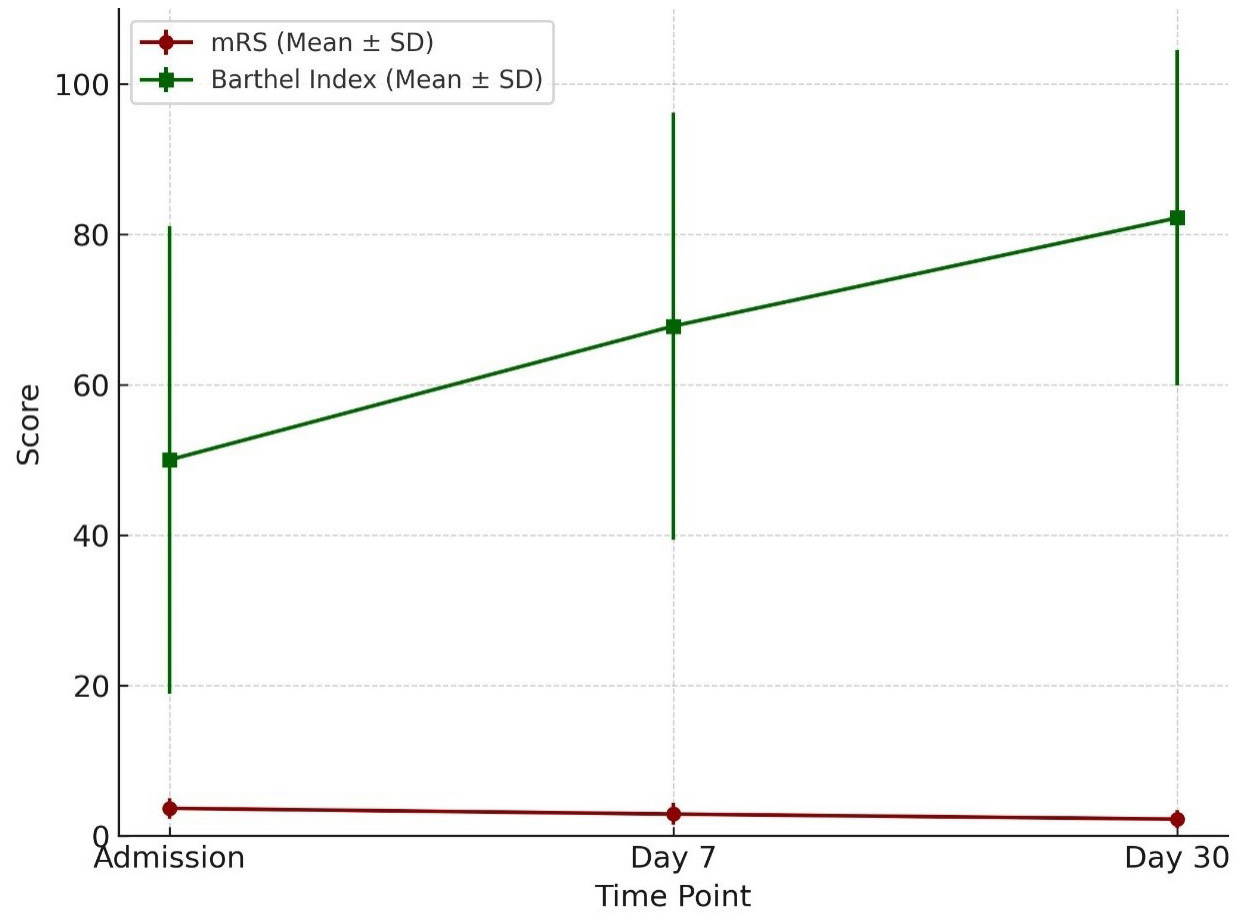
Functional outcome over time Functional outcome trajectories over 30 days. The Barthel Index (mean ± SD) showed a progressive increase from admission to day 30, reflecting improved functional independence. Conversely, the modified Rankin Scale (mRS) (mean ± SD) demonstrated a gradual decline, consistent with reduced disability severity over time.

Modified Rankin Scale (mRS) scores declined progressively from admission to day 30, while Barthel Index scores increased steadily over the same period. By day 30, the change in Barthel Index scores reached statistical significance. Importantly, variation in scores across participants was observed (Table 12 and Figure 9).

**Table 12:** Outcomes variables.

**Variables**	**Mean (SD)**	**Median (IQR)**	**Minimum**	**Maximum**	**Shapiro-Wilk *P*-value**
**Outcomes**					
**mRS**					
At admission	3.67 (1.34)	4 (2)	1	5	< 0.001
At 7 days	2.90 (1.43)	3 (3)	1	5	< 0.001
At 30 days	2.23 (1.19)	2 (2)	1	4	< 0.001
**Barthel Index**					
At admission	50 (31.1)	50 (47.5)	0	100	0.07
At 7 days	67.8 (28.4)	65 (45)	10	100	0.29
At 30 days	82.2 (22.3)	90 (26.3)	30	100	0.001

mRS, modified Rankin Scale.

Initial blood pressure readings were generally elevated. Mean systolic and diastolic pressures at presentation were high and demonstrated wide variability over the first 24 h. Standard deviations and coefficients of variation for both systolic and diastolic pressures indicated moderate dispersion. Daytime and nighttime average blood pressure values were similar; however, the calculated day/night ratio and dipping percentages showed that most patients had a non-dipping or reverse-dipping pattern, particularly for systolic pressure (Supplementary Table 2).

**Figure 10:**
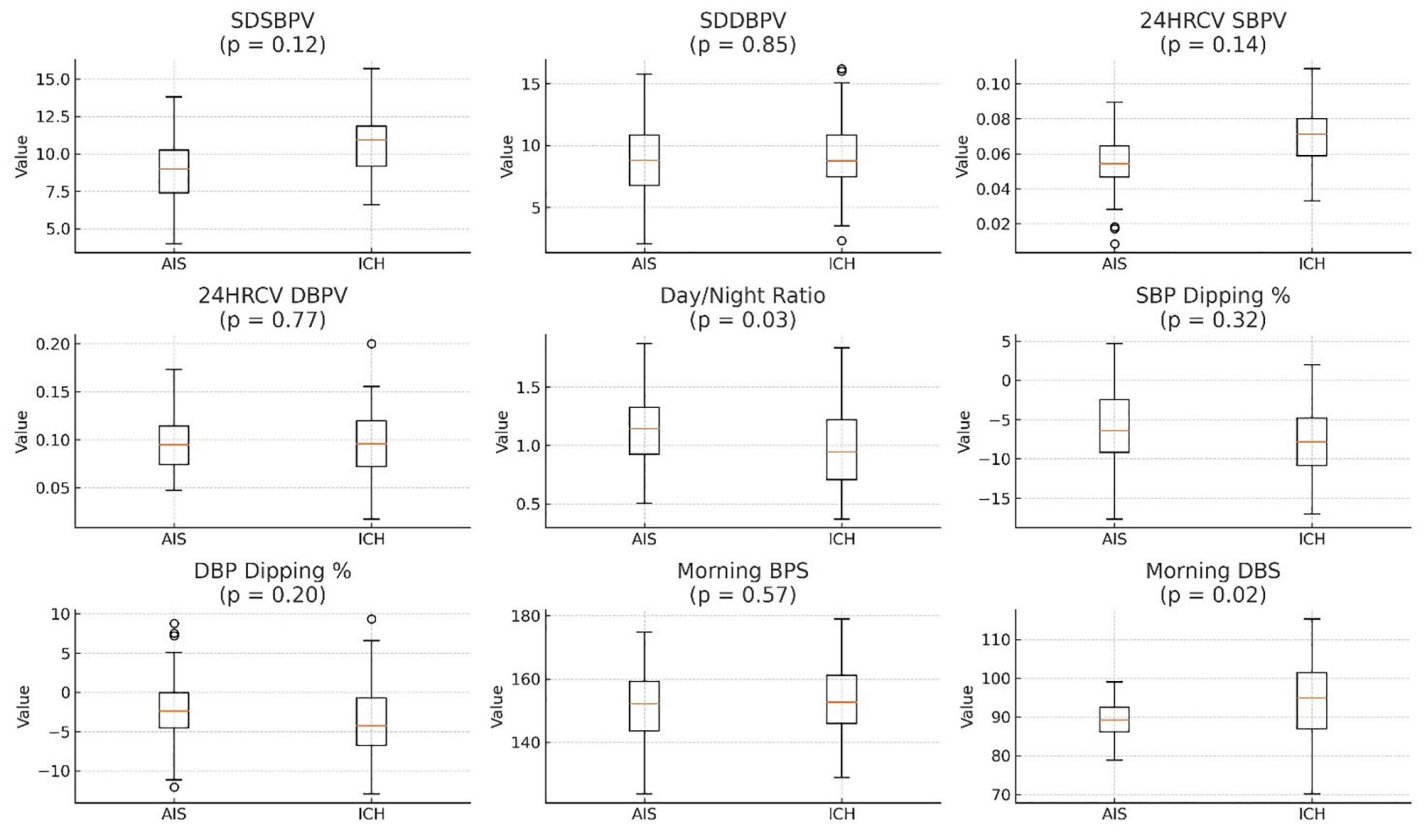
ICH generally has higher BP variability (SDSBPV, SDDPBV, and 24HRCV) compared to AIS. Day/Night ratio is slightly lower in ICH. SBP Dipping % is negative for both AIS and ICH, with AIS showing a slightly greater dip. SDSBPV, Standard Deviation of Systolic Blood Pressure Variability over 24 h; SDDBPV, Standard Deviation of Diastolic Blood Pressure Variability over 24 h; 24HRCV_SBPV, 24-h Coefficient of Variation of Systolic Blood Pressure; 24HRCV_DBPV, 24-H Coefficient of Variation of Diastolic Blood Pressure; Day/Night Ratio, Ratio of average daytime to nighttime BP (values >1 suggest non-dipping); SBP Dipping %, Percentage decrease in systolic BP at night compared to day (negative = dipping); DBP Dipping %, Percentage decrease in diastolic BP at night compared to day (negative = dipping); Morning BPS, Morning Systolic Blood Pressure (measured between 6–10 AM); Morning DBS, Morning Diastolic Blood Pressure (measured between 6–10 AM), AIS, Acute Ischaemic Stroke; ICH, Intra-Cerebral Hemorrhage.

Morning blood pressure values were higher in patients with ICH, with greater median values and wider interquartile ranges for both systolic and diastolic measurements. Comparisons between AIS and ICH patients showed generally higher blood pressure variability measures (e.g., SDSBPV, 24HRCV_SBPV) in the ICH group, although not all differences were statistically significant. The day/night ratio was slightly lower in the ICH group, while both groups exhibiting negative dipping percentages (Supplementary Table 3 and Figure 10).

### 3.2 Univariable analysis

The univariable analyses revealed that neurological severity, as measured by the NIHSS, was a robust predictor of stroke outcome. Higher scores at admission, 24 h, and 7 days were significantly associated with worse mRS and lower Barthel Index scores. GCS also emerged as a relevant marker, with lower scores at admission and day 7 correlating with greater functional disability.

Blood pressure patterns such as the day/night ratio showed statistically significance in univariable analysis, suggesting potential relevance of circadian BP patterns in prognosis. However, more traditional BP measures (e.g., initial SBP or mean BP) did not reach statistical significance.

Sociodemographic variables such as age, sex, marital status, and educational level similarly did not demonstrate significant associations with functional outcome at day 7 and 30, though patients with tertiary education showed better recovery by day 30. Ethnicity, residential location, and lifestyle factors like alcohol and tobacco use did not significantly influence functional recovery.

Stroke subtype analysis indicated a trend toward better outcomes in AIS compared to ICH, though this did not achieve statistical significance. Within ischemic stroke, patients with small-vessel occlusion had more favorable outcomes than those with large-artery atherosclerosis, particularly by day 30. The limited number of cardioembolic strokes precluded meaningful analysis.

No clear association was found between therapeutic interventions such as thrombolysis or head positioning and patient outcomes, likely due to the small number of patients receiving these treatments. Use of antihypertensive therapy trended toward better outcomes but did not reach statistical significance (Tables 13, 14).

**Table 13:** Significant independent samples *T*-test Results for mRS at 30 days.

**Variable**	***p*-value**	**95% CI**
NIHSS score at admission	< 0.001	−16.49 to −7.73
NIHSS score at 24 h	< 0.001	−15.44 to −7.50
NIHSS score at 7 days	< 0.001	−13.67 to −7.30
GCS at admission	0.007	0.43 to 2.64
GCS at 7 days	0.033	0.07 to 1.60
Day/night ratio	0.019	0.05 to 0.58

NIHSS, National Institute of Health Stroke Scale; GCS, Glasgow Coma Scale.

**Table 14:** Significant independent samples *T*-test results for Barthel Index at 30 days.

**Variable**	***p*-value**	**95% CI range**
NIHSS score at admission	< 0.001	6.12 to 15.15
NIHSS score at 24 h	< 0.001	6.96 to 14.80
NIHSS score at 7 days	< 0.001	6.24 to 12.79
GCS at admission	0.01	−2.51 to −0.35
GCS at 24 h	0.03	−2.34 to 0.55

NIHSS, National Institute of Health Stroke Scale; GCS, Glasgow Coma Scale.

### 3.3 Multivariable analysis

**Figure 11:**
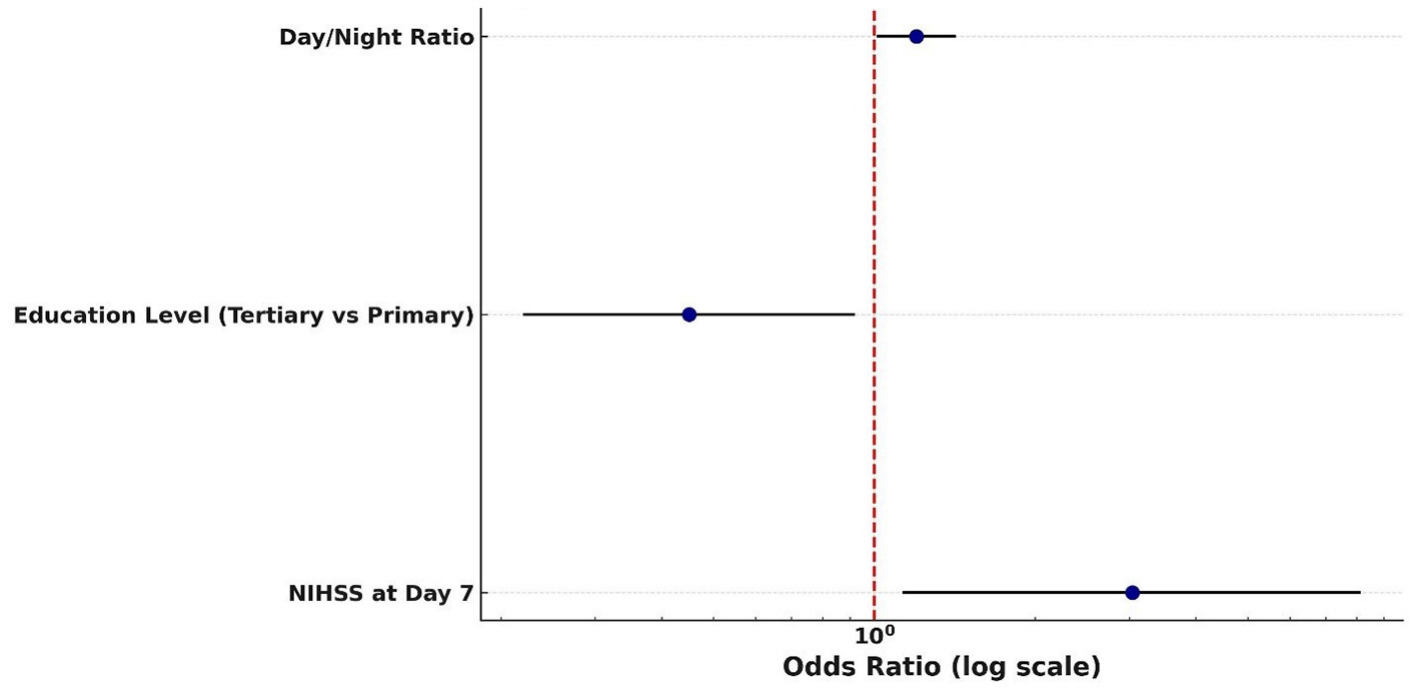
Predictors of poor 30-day outcome (mRS 3–5) Forest plot of predictors of poor functional outcome (mRS 3–5) at 30 days. Odds ratios (with 95% confidence intervals) are displayed on a logarithmic scale for day/night blood pressure ratio, education level (tertiary vs. primary), and NIHSS score at day 7. The red dashed line indicates the null value (OR = 1).

In the logistic regression models for mRS, the NIHSS score at day 7 emerged as the only statistically significant independent predictor of poor outcome, with an odds ratio suggesting a threefold increase in risk per unit increase in score. Other variables, including admission and 24-h NIHSS, GCS, and BP variability indices, failed to retain significance, possibly due to insufficient power (Table 15 and Figure 11).

**Table 15:** Model coefficients—mRS.

**Predictor**	***p*-value**	**Odds ratio**	**95% CI range**
NIHSS score at admission	0.32	1.35	0.75 to 2.44
NIHSS score at 24 h	0.11	0.43	0.15 to 1.22
NIHSS score at 7 days	0.03	3.04	1.13 to 8.15
GCS at admission	0.997	< 0.001	Not estimable
GCS at 7 days	0.998	>10,000	Not estimable
Day/night ratio	0.16	0.08	0.002 to 2.84

NIHSS, National Institute of Health Stroke Scale; GCS, Glasgow Coma Scale.

Similarly, in models predicting Barthel Index outcomes, the NIHSS score at 7 days approached statistical significance, with lower scores trending toward better recovery. However, this finding did not meet the conventional threshold for significance. GCS values again showed statistical instability, undermining their interpretability in the multivariable context (Table 16).

**Table 16:** Model coefficients—Barthel Index.

**Predictor**	***p*-value**	**Odds ratio**	**95% CI range**
NIHSS score at admission	0.67	1.05	0.839 to 1.31
NIHSS score at 24 h	0.77	1.06	0.707 to 1.60
NIHSS score at 7 days	0.10	0.66	0.409 to 1.07
GCS at admission	0.995	>10,000	Not estimable
GCS at 24 h	0.995	< 0.001	Not estimable

NIHSS, National Institute of Health Stroke Scale; GCS, Glasgow Coma Scale.

## 4 Discussion

The findings revealed that the proportion of hemorrhagic strokes in this cohort (56.3%) was notably high compared to high income countries, where ischemic strokes predominate. This observation aligns with findings from other Sub-Saharan African settings, where uncontrolled hypertension, limited awareness, and delayed hospital presentation are major contributors to intracerebral hemorrhage. The exceptionally high prevalence of hypertension in this study (91.5%) further supports its central role in the occurrence and subtype distribution of stroke in this context.

### 4.1 Blood pressure variability patterns in ischemic stroke (AIS) and hemorrhagic stroke (ICH)

Beyond subtype distribution, this study demonstrated distinct differences in blood pressure variability (BPV) between ischemic (AIS) and hemorrhagic (ICH) strokes. In AIS patients, the mean systolic BP variability (SDSBPV) was 8.65 mmHg and diastolic BP variability (SDDPBV) was 9.0 mmHg, with corresponding coefficients of variation (CV) of 5.6% and 9.59%, respectively, indicating moderate fluctuations. The mean day/night systolic BP ratio in AIS was 1.11 (median 0.94), reflecting preserved but attenuated circadian rhythm, although nocturnal systolic and diastolic dipping was blunted at −6.31% and −3.13%, respectively.

By contrast, ICH patients exhibited greater BP variability and more profound circadian disruption. Their mean SDSBPV was higher at 10.5 mmHg (median 10.9 mmHg), and diastolic variability showed a wider range (mean 9.5 mmHg; range 1.4–21.2 mmHg). The CVs were 7% for systolic and 10% for diastolic BP, suggesting heightened sympathetic activity and baroreflex impairment. The average day/night BP ratio in ICH was significantly lower than AIS (0.999 vs. 1.11, *p* = 0.03), with individual ratios reaching as high as 2.48. Nocturnal systolic dipping was similar to AIS (−6.15%, median −5.5%), but diastolic dipping was even more attenuated (median −1.75%), consistent with a “non-dipping” or “reverse-dipping” profile.

These BPV abnormalities likely result from the combined effects of chronic hypertension, acute neurological injury, and systemic stress responses, all of which exacerbate autonomic dysregulation. While both stroke subtypes exhibit impaired circadian regulation, this disruption appears more pronounced in hemorrhagic strokes. This may be attributed to the acute elevation in intracranial pressure, hematoma expansion, and more profound sympathetic nervous system activation that typically accompany hemorrhagic strokes. These physiological disturbances can severely impair baroreflex sensitivity and disrupt central autonomic pathways, including those involving the hypothalamus and brainstem, which are critical for maintaining circadian blood pressure rhythms. Moreover, the sudden mechanical and inflammatory insult to brain tissue in ICH may result in greater autonomic dysregulation compared to the more ischemic, metabolically driven injury in AIS (Yperzeele et al., 2015). Prior studies support these findings. Ohkubo et al. (2002) and Qureshi et al. (2010) reported similar non-dipping profiles and elevated BPV as markers of autonomic dysfunction and poor prognosis, particularly in hemorrhagic strokes. Sakamoto et al. (2013) further highlighted BPV as a contributor to hematoma expansion in ICH, reinforcing its clinical relevance.

### 4.2 Association between blood pressure variability and functional outcome

Functional outcomes assessed through mRS and Barthel Index revealed progressive recovery over the 30-day period. The mRS improved from a mean of 3.67 at admission to 2.90 at day 7, while the Barthel Index rose from 50 at admission to 82.2 by day 30 (*p* = 0.001). The Barthel Index demonstrated greater sensitivity to incremental improvements in independence, complementing the global disability measure captured by the mRS. This dual-assessment approach is in line with prior recommendations by Quinn et al. (2009) and Wolfe et al. (2011). The combined use provides a more comprehensive understanding of patient outcomes. Quinn's review emphasized the BI's strength in measuring activities of daily living, while noting its limitations such as ceiling effects and inconsistent versions across trials and advocated for its standardization alongside mRS to boost accuracy and consistency. Wolfe's study refined this approach by establishing BI score thresholds that corresponded to specific mRS grades, enhancing predictive power through sensitivity and specificity analyses. Together, these findings demonstrate that each scale captures distinct dimensions of disability (i.e., BI for functional independence and mRS for global disability) thus improving outcome precision and informing more nuanced post-stroke recovery evaluations.

Despite the observed BPV elevations, most variability metrics including SDSBPV, SDDPBV, and CV were not independently associated with functional outcomes in either univariable or multivariable models. The key exception was the day/night BP ratio, which significantly predicted mRS scores (*p* = 0.02, 95% CI: 0.05–0.58). This suggests that disrupted circadian rhythms, more than absolute BP variability, may have prognostic significance. One likely reason is that the day/night blood pressure ratio reflects underlying circadian rhythm integrity, which plays a pivotal role in cardiovascular and neurological regulation. Unlike standard BP variability metrics (e.g., SD or CV), the day/night ratio captures temporal patterns specifically, the expected nocturnal dip in BP during sleep. When this dip is blunted or reversed (a “non-dipping” or “riser” profile), it often signals autonomic dysfunction, vascular stiffness, or impaired neurohormonal control, all of which are linked to poorer outcomes after stroke (Gumz et al., 2023). In contrast, metrics like SDSBPV or CV quantify amplitude of fluctuation, but not timing, making them less sensitive to the pathophysiological processes tied to stroke recovery. Mori et al. (2012) similarly found that circadian BP abnormalities were associated with early neurological deterioration and poorer outcomes.

Neurological severity, measured by the NIH Stroke Scale (NIHSS), emerged as the strongest predictor of functional outcome. The NIHSS score at 7 days was independently associated with poor mRS scores (*p* = 0.03, OR = 3.04, 95% CI: 1.13–8.15). This could be because it reflects stabilized neurological recovery, incorporating both early clinical improvement and therapeutic responses to acute interventions. Unlike baseline or 24-h scores, the day 7 assessment captures the resolution of transient factors such as cerebral edema or sedation and offers a clearer representation of residual deficits. Additionally, it is less influenced by post-discharge confounders and shows superior statistical sensitivity, making it a practical and reliable prognostic tool during hospitalization. Tudor et al. (2020), Kerr et al. (2012), and Janardhan et al. (2015) reported that NIHSS scores at both admission and 24 h were significant predictors in univariable models (*p* < 0.001), but their significance was lost in multivariable analysis because of collinearity. This is consistent with the methodological challenges of multicollinearity, where highly correlated predictors (e.g., sequential NIHSS scores) share overlapping explanatory variance (Gregorich et al., 2021). Such redundancy reduces statistical independence, making later or adjusted measures such as day 7 NIHSS in our study more robust predictors of functional outcome. The Glasgow Coma Scale (GCS) at admission (*p* = 0.01) and on day 7 (*p* = 0.03) exhibited comparable trends, achieving significance in univariable analyses but not retaining independent predictive value in multivariable models, most likely due to collinearity (a high degree of correlation between covariates that obscures their unique contributions with NIHSS. Early NIHSS measurements (e.g., at baseline or 24 h) may be confounded by transient factors such as cerebral edema, reperfusion injury, or sedation. By day 7, these influences typically abate, providing a more reliable indicator of sustained neurological recovery.

The lack of consistent, independent associations between most BPV indices and functional outcomes suggests that BPV may be more of a secondary marker reflecting stroke severity, impaired autoregulation, or autonomic instability, rather than a primary determinant of outcome. This aligns with findings by Sandset et al. (2011) conducted within the IST-3 trial cohort, BPV during the first 24 h after ischemic stroke was initially associated with poor functional outcomes at 6 months. However, this association weakened after adjusting for stroke severity, suggesting that BPV may be more of a marker of underlying neurological injury rather than an independent prognostic factor.

### 4.3 Association of other covariates and functional outcome

Interestingly, education level, specifically tertiary education, was independently associated with better 30-day outcomes (*p* = 0.03). This underscores the role of health literacy, rehabilitation adherence, and socioeconomic factors in stroke recovery. Tertiary education independently contributes to improved short-term clinical outcomes through a combination of enhanced health literacy, proactive patient engagement, and effective communication with healthcare providers. These individuals often access care earlier, navigate health systems more skillfully, and benefit from socioeconomic advantages such as better insurance, nutrition, and living conditions, all of which support recovery. With a statistically significant association, the data suggests that educational attainment itself plays a direct, measurable role in influencing post-treatment prognosis, even after controlling for confounding variables. Other demographic and clinical factors, such as age, sex, stroke subtype, thrombolysis, and antihypertensive therapy, did not show significant associations with outcome. Imaging characteristics and neuroanatomical lesion data were not fully integrated into the outcome analysis due to inconsistent acquisition protocols, incomplete lesion descriptions, and logistical barriers to standardized data collection. Many scans lacked uniform timing or resolution, and lesions were poorly annotated, limiting their usefulness for quantitative modeling. Additionally, technical and analytical limitations like missing data and variability in segmentation prevented reliable integration into statistical outcome frameworks., thus representing a potential area for future multimodal studies.

This study also highlights two critical timing challenges in acute stroke care. The median time from last known well to arrival was approximately 15 h for AIS and 13 h for ICH, with a larger proportion of ICH patients reaching the hospital within the first 24 h. This difference is likely explained by the more dramatic clinical presentation of ICH, which prompts urgent medical attention, compared to the often more subtle and fluctuating symptoms of AIS that may be underestimated or misinterpreted. In the Tanzanian context, limited public awareness, reliance on traditional remedies, poor emergency transport, and geographic barriers further contribute to delayed hospital presentation, particularly for ischemic stroke. These prolonged delays observed restrict opportunities for acute interventions, particularly reperfusion therapies and highlight the need for community education, strengthened pre-hospital systems, and rapid referral pathways to improve early stroke care. Such late presentation, also reported in other sub-Saharan African studies, emphasizes the urgent need for community awareness campaigns, better referral networks, and strengthened emergency transport systems.

In addition, ambulatory blood pressure monitoring (ABPM) was initiated on average 13.5 h after hospital arrival, with a wide range from 1 to 26 h. Unlike the clustered pre-hospital delays, the start of ABPM was more evenly distributed across the first 24 h, reflecting variations in clinical workflow and patient stabilization. Importantly, this delay in initiating monitoring may have led to an underestimation of very early BPV, as fluctuations occurring immediately after stroke onset and after admission might not have been captured.

### 4.4 Comparison of functional outcomes: mRS vs. Barthel Index

In our study, both the modified Rankin Scale (mRS) and Barthel Index were used to evaluate functional outcomes following stroke. The mRS captured global disability meanwhile, the Barthel Index on the other hand measures independence in activities of daily living.

**Figure 12:**
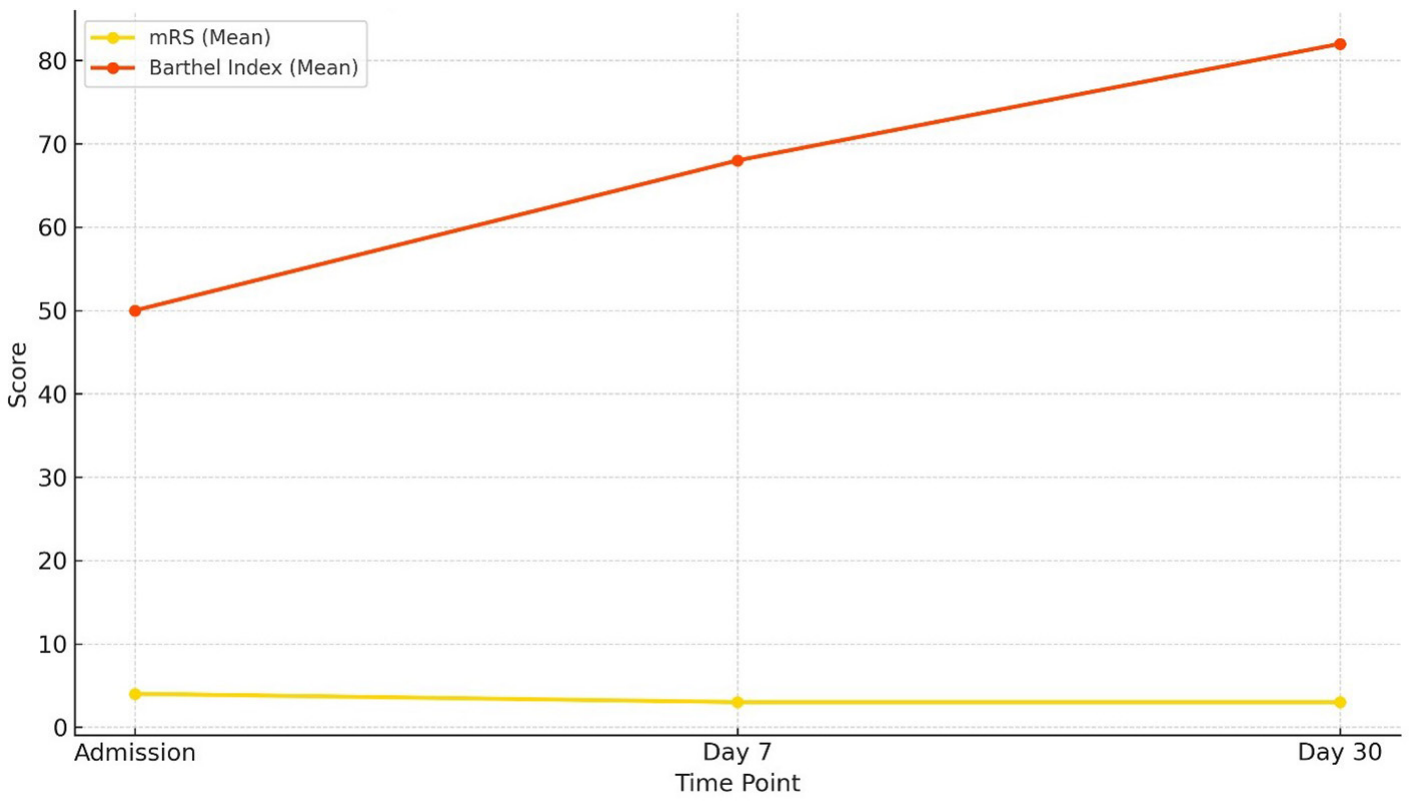
Comparison of functional status: mRS vs. Barthel Index Changes in functional outcomes over 30 days. The Barthel Index (mean) increased steadily from admission to day 30, indicating progressive improvement in functional independence, while the modified Rankin Scale (mRS) (mean) showed a gradual decline, consistent with reduced disability severity.

While both tools showed improvement in functional status, the Barthel Index appeared more sensitive in detecting incremental recovery over a 30-day period, likely due to its emphasis on physical independence. The mRS, being more categorical and subjective, may be better suited for broader outcome classification (Figure 12).

The distinction between the Barthel Index (BI) and the modified Rankin Scale (mRS) has been well established in the stroke literature. Wolfe et al. (1991) first emphasized that the BI is particularly responsive to functional gains achieved through rehabilitation, whereas the mRS is more suited to capturing overall disability status. This has been supported by subsequent studies. Uyttenboogaart et al. (2007) showed that at 3 months post-stroke, nearly 40% of patients graded as mRS 1–2 still demonstrated residual dependency in at least one activity of daily living (ADL) according to the BI, while about 30% of those rated mRS 3 required walking assistance, highlighting the BI's greater sensitivity in detecting ADL limitations that may be masked by the mRS. Conversely, Cioncoloni et al. (2012) demonstrated that the discriminatory power of the mRS improves with time; at 6 months post-stroke, it more clearly separated functional independence (grades 0–2) from dependence (grades 3–5), paralleling BI classifications. Together, these findings show the complementary strengths of the two scales: the BI excels in capturing incremental improvements in day-to-day functioning, especially in the acute and subacute phases, while the mRS provides a more global perspective on long-term disability. For this reason, the dual use of both instruments in stroke outcome research offers a more nuanced and comprehensive assessment of patient recovery trajectories.

This dual assessment approach is supported by previous literature. Quinn et al. (2009) emphasized the complementary value of using both tools in stroke trials, with mRS correlating more with quality of life and Barthel Index providing detailed insights into daily functional independence.

Thus, our findings align with existing research and reinforce the utility of combining both mRS and Barthel Index in evaluating short-term outcomes post-stroke. The concordance between both tools also enhances confidence in the functional improvement observed in this cohort.

### 4.5 Study limitations

Several other limitations should be acknowledged. The modest sample size (*n* = 48) limited statistical power and may have increased the likelihood of type II errors, particularly in detecting subtle associations between BPV and outcomes. The cohort was drawn from two urban tertiary hospitals, potentially limiting generalizability to rural populations. The 30-day follow-up period precludes conclusions about long-term outcomes or mortality. Potential confounding factors, including lesion size, infarct location, and medication regimens, were not analyzed in detail, and medication effects on BPV were not controlled for. While neuroimaging data were collected, they were not integrated into prognostic modeling an area for future enhancement. Additionally, some functional outcome assessments at day 30 were conducted via telephone, which may have introduced measurement bias or misclassification, particularly for more nuanced aspects of disability captured by the Barthel Index or mRS. Although telephone assessments are commonly used in stroke research, their validity may vary depending on respondent reliability and patient comprehension, potentially influencing accuracy. Nonetheless, this study offers novel insights into BPV and stroke outcomes in an underrepresented African cohort, with the combined use of mRS and Barthel Index providing a nuanced assessment of functional recovery.

Overall, the findings reinforce that while BPV, particularly circadian disruption, is associated with physiological instability post-stroke, NIHSS remains the most robust and independent predictor of functional recovery. The day/night BP ratio may offer an additional, easily derived prognostic marker from 24-h ABPM, warranting further exploration. These results align with Rothwell et al. (2010), who identified short-term BPV as a predictor of early recurrence, though differences in sample size and monitoring duration may explain inconsistencies in predictive strength across studies.

### 4.6 Recommendations

Based on these findings, several clinical recommendations can be made. First, 24-h ABPM should be incorporated into acute stroke management protocols, especially for ICH patients, where BP instability is more pronounced. Stratified BP control strategies by stroke subtype may help mitigate risk tighter control in ICH may reduce the risk of hematoma expansion. Routine NIHSS scoring at admission, 24 h, and day 7 should be adopted, given its consistent prognostic utility. Health systems, especially in resource-limited settings like Tanzania, should invest in validated 24-h BP monitoring technologies and train healthcare workers in interpreting BPV metrics and circadian patterns.

For future research, larger multicenter studies are needed to confirm the prognostic utility of BPV, particularly the day/night BP ratio, and to explore its interaction with stroke mechanisms and long-term outcomes. Mechanistic investigations focusing on autonomic dysfunction, cerebral autoregulation, and inflammatory pathways may further elucidate how BPV interacts with stroke pathophysiology.

## 5 Conclusion

In this Tanzanian cohort of acute stroke patients, BPV in the first 24 h was characterized by elevated morning pressures, reduced nocturnal dipping, and non-dipping or reverse-dipping patterns more pronounced in hemorrhagic strokes. However, BPV measures did not show a significant association with functional outcomes at 30 days. Instead, neurological severity (NIHSS at 7 days) emerged as the most consistent predictor of poor recovery. Lower educational attainment also appeared to influence functional outcomes. Both the modified Rankin Scale and Barthel Index proved valuable for assessing recovery, with the Barthel Index demonstrating greater sensitivity to subtle improvements. These findings underscore the prognostic importance of early neurological assessment and highlight the potential influence of socioeconomic factors on stroke rehabilitation.

## Data Availability

The raw data supporting the conclusions of this article will be made available by the authors, without undue reservation.
