# Supplementary material for: Impact of short-term (24 h) blood pressure variability on 30-days clinical outcomes of acute strokes at two tertiary hospitals in Dar-es-Salaam
Source: Front Stroke. 2025 Nov 11;4:1700321. doi: 10.3389/fstro.2025.1700321 (PMC12802666; doi:10.3389/fstro.2025.1700321)
Supplement: Supplementary file 1 [file Table_1.docx]

APPENDIX

**TABLES**

**Table 1: Demographic Variables of Study Participants**

| **Variable** | **Category** | **Frequency (n = 48) (%)** |
| --- | --- | --- |
| **Age Groups (years)** | 18-32 | 5 (10.4) |
|  | 33-46 | 9 (18.9) |
|  | 47-60 | 19 (39.6) |
|  | >60 | 15 (31.3) |
| **Sex** | Male | 31 (64.6) |
|  | Female | 17 (35.4) |
| **Residence** | Dar-es-Salaam | 42 (89.4) |
|  | Another region | 5 (10.6) |
| **Educational Level** | Primary | 27 (57.4) |
|  | Secondary | 14 (29.8) |
|  | Tertiary | 6 (12.8) |
| **Marital Status** | Married | 32 (66.7) |
|  | Single | 6 (12.5) |
|  | Widowed | 4 (8.3) |
|  | Separated | 6 (12.5) |
| **Ethnicity** | Black African | 46 (95.8) |
|  | Asian | 2 (4.2) |
| **Alcohol Intake** | Yes | 13 (27.1) |
| **Current Smoking** | Yes | 13 (27.1) |
| **Comorbidities** | Hypertension | 43 (91.5) |
|  | Diabetes Mellitus | 3 (6.4) |
|  | Dyslipidemia | 1 (2.1) |
|  | Chronic Kidney Disease | 0 (0.0) |
|  | Atrial Fibrillation | 0 (0.0) |
|  | Previous Stroke | 1 (2.1) |
|  | Previous Ischemic H/O Heart Disease | 1 (2.1) |

**Table 14:** **Primary exposure Variables**

| **Variables** | **Mean (SD)** | **Median (IQR)** | **Minimum** | **Maximum** | **Shapiro-Wilk**  **P-Value** |
| --- | --- | --- | --- | --- | --- |
| **PRIMARY EXPOSURE** |  |  |  |  |  |
| Initial SBP | 171 (27.5) | 176 (27) | 118 | 212 | <0.001 |
| Initial DBP | 102 (16.1) | 103 (16.5) | 70 | 134 | 0.004 |
| SDSBPV | 9.6 (4.03) | 10.4 (6.65) | 1.2 | 15.7 | 0.023 |
| SDDPBV | 9.25 (4.53) | 9.2 (5.9) | 1.4 | 21.2 | 0.32 |
| Mean SBP | 153 (14.4) | 154 (18) | 110 | 180 | 0.62 |
| Mean DBP | 98 (7.95) | 98.5 (9.25) | 71 | 110 | 0.03 |
| 24HRCV_SBPV | 6.33 (2.66) | 6.79 (4.37) | 0.745 | 11.0 | 0.08 |
| 24HRCV_DBPV | 9.62 (5.05) | 9.48 (6.25) | 1.38 | 22.1 | 0.34 |
| Daytime | 10 (2.76) | 9.6 (4.13) | 5.1 | 14.7 | 0.16 |
| Nighttime | 10.5 (2.84) | 10.6 (3.97) | 5.4 | 15.9 | 0.24 |
| Morning BPS | 153 (16.82) | 153 (24.0) | 108 | 179 | 0.181 |
| Morning DBS | 91 (9.042) | 91 (9.0) | 62 | 123 | <0.001 |
| Day/Night Ratio | 1.04 (0.46) | 0.911 (0.42) | 0.354 | 2.48 | 0.002 |
| SBP Dipping % | -6.17 (8.84) | -7.05 (15.6) | -20 | 10 | 0.03 |
| DBP Dipping % | -3.28 (8.3) | -3 (12.4) | -19.5 | 9.7 | 0.10 |

SDSBPV – Standard Deviation of Systolic Blood Pressure Variability over 24 hours;
SDDBPV – Standard Deviation of Diastolic Blood Pressure Variability over 24 hours;
24HRCV_SBPV – 24-Hour Coefficient of Variation of Systolic Blood Pressure;
24HRCV_DBPV – 24-Hour Coefficient of Variation of Diastolic Blood Pressure;
Day/Night Ratio – Ratio of average daytime to nighttime BP (values >1 suggest non-dipping);
SBP Dipping % – Percentage decrease in systolic BP at night compared to day (negative = dipping); DBP Dipping % – Percentage decrease in diastolic BP at night compared to day (negative = dipping); Morning BPS – Morning Systolic Blood Pressure (measured between 6–10 AM); Morning DBS – Morning Diastolic Blood Pressure (measured between 6–10 AM), AIS-Acute Ischemic Stroke; ICH- Intra-Cerebral Hemorrhage

**Table 15:** **Blood pressure variability and pattern in AIS vs ICH**

| **Stroke Subtype** | **SDSBPV** | **SDDBPV** | **24HRCV**  **SBPV** | **24HRCV**  **DBPV** | **Day/Night Ratio** | **SBP Dipping %** | **DBP Dipping %** | **Morning BPS** | **Morning DBS** |
| --- | --- | --- | --- | --- | --- | --- | --- | --- | --- |
| Mean |  |  |  |  |  |  |  |  |  |
| AIS | 8.65 | 9.00 | 5.66 | 9.59 | 1.11 | -6.31 | -3.13 | 152 | 89.2 |
| ICH | 10.5 | 9.50 | 6.95 | 9.69 | 0.999 | -6.15 | -3.57 | 153 | 92 |
| Median |  |  |  |  |  |  |  |  |  |
| AIS | 9.10 | 8.60 | 5.95 | 9.05 | 0.94 | -7.70 | -3.70 | 153 | 89 |
| ICH | 10.9 | 9.60 | 7.15 | 9.71 | 0.91 | -5.50 | -1.75 | 154 | 93 |
| Minimum |  |  |  |  |  |  |  |  |  |
| AIS | 1.20 | 1.50 | 0.745 | 1.40 | 0.50 | -20.00 | -15.40 | 116 | 69 |
| ICH | 3.40 | 1.40 | 2.04 | 1.38 | 0.35 | -19.60 | -19.50 | 108 | 62 |
| Maximum |  |  |  |  |  |  |  |  |  |
| AIS | 14.9 | 18.3 | 9.73 | 20.80 | 2.10 | 10.00 | 9.40 | 176 | 99 |
| ICH | 15.7 | 21.2 | 11.00 | 22.10 | 2.48 | 9.80 | 9.70 | 179 | 123 |
| p-value |  |  |  |  |  |  |  |  |  |
| AIS | 0.12 | 0.86 | 0.14 | 0.77 | 0.03 | 0.32 | 0.199 | 0.57 | 0.02 |
| ICH | 0.17 | 0.50 | 0.48 | 0.63 | 0.01 | 0.049 | 0.14 | 0.44 | 0.002 |

SDSBPV – Standard Deviation of Systolic Blood Pressure Variability over 24 hours;
SDDBPV – Standard Deviation of Diastolic Blood Pressure Variability over 24 hours;
24HRCV_SBPV – 24-Hour Coefficient of Variation of Systolic Blood Pressure;
24HRCV_DBPV – 24-Hour Coefficient of Variation of Diastolic Blood Pressure;
Day/Night Ratio – Ratio of average daytime to nighttime BP (values >1 suggest non-dipping);
SBP Dipping % – Percentage decrease in systolic BP at night compared to day (negative = dipping); DBP Dipping % – Percentage decrease in diastolic BP at night compared to day (negative = dipping); Morning BPS – Morning Systolic Blood Pressure (measured between 6–10 AM); Morning DBS – Morning Diastolic Blood Pressure (measured between 6–10 AM), AIS-Acute Ischemic Stroke; ICH- Intra-Cerebral Hemorrhage

**Appendix 1: Functional Status Questionnaire**

**The Standard Modified Rankin Scale (mRS)**

To be used when there is direct contact with the patient

The scale runs from 0–6, running from perfect health without symptoms to death.

0 - No symptoms.

1 - No significant disability. Able to carry out all usual activities, despite some symptoms.

2 - Slight disability. Able to look after own affairs without assistance, but unable to carry out all previous activities.

3 - Moderate disability. Requires some help, but able to walk unassisted.

4 - Moderately severe disability. Unable to attend to own bodily needs without assistance, and unable to walk unassisted.

5- Severe disability: bed-ridden, incontinent, and requiring constant nursing care and attention.

6- Dead.

**Interpretation**

- Good functional outcome: 0-3
- Poor functional outcome: 4-6

**Appendix 2: Revised simplified modified Rankin Scale questionnaire**

To be used for patients that are being followed up by telephone

- If you had to, could you live alone without any help from another person? This means being able to bathe, use the toilet, shop, prepare or get meals, and manage finances. (score ≤2 or ≥3)

(**Swahili**: Je ungekua nauwezo, ungeweza kuishi mwenye bila mtu? Kwa maana ya kujitegemea kuoga, kutumia vyo, kwenda dukani, kuandaa/kupika chakula, kuendesha huduma za kiuchumi)

- Can you do everything that you were doing right before your stroke, even if slower and not as much? (Score 2 or ≤1)

(**Swahili:** Je unaweza kufanya kila kitu ulichokua unaweza kufanya kabla hujapata kiharusi, hata kama sio kwa kasi ile ile?)

- Are you completely back to the way you were right before your stroke? (Score 0 or 1)

(**Swahili:** Je hali yako imerudi kama ilivyokua kabla hujapata kiharusi?)

- Can you walk from one room to another without help from another person? (Score 3 or ≥4)

(**Swahili:** Je, unaweza kutembea kutoka chumba kimoja hadi kingine bila msaada wa mtu mwingine?)

- Can you sit up in bed without any help? OR are you bedridden or needing constant supervision? (Score 4 or 5)

(**Swahili:** Je, unaweza kukaa kitandani mwenyewe bila msaada wowote? Au huwezi kutoka kitandani, au kuhitaji msaada wakati wote?)

- Deceased (6)

(**Swahili:** Amefariki)

**Interpretation**

- Good functional outcome: 0-3
- Poor functional outcome: 4-6

**Appendix 3: Pictogram aid for mRS scoring**


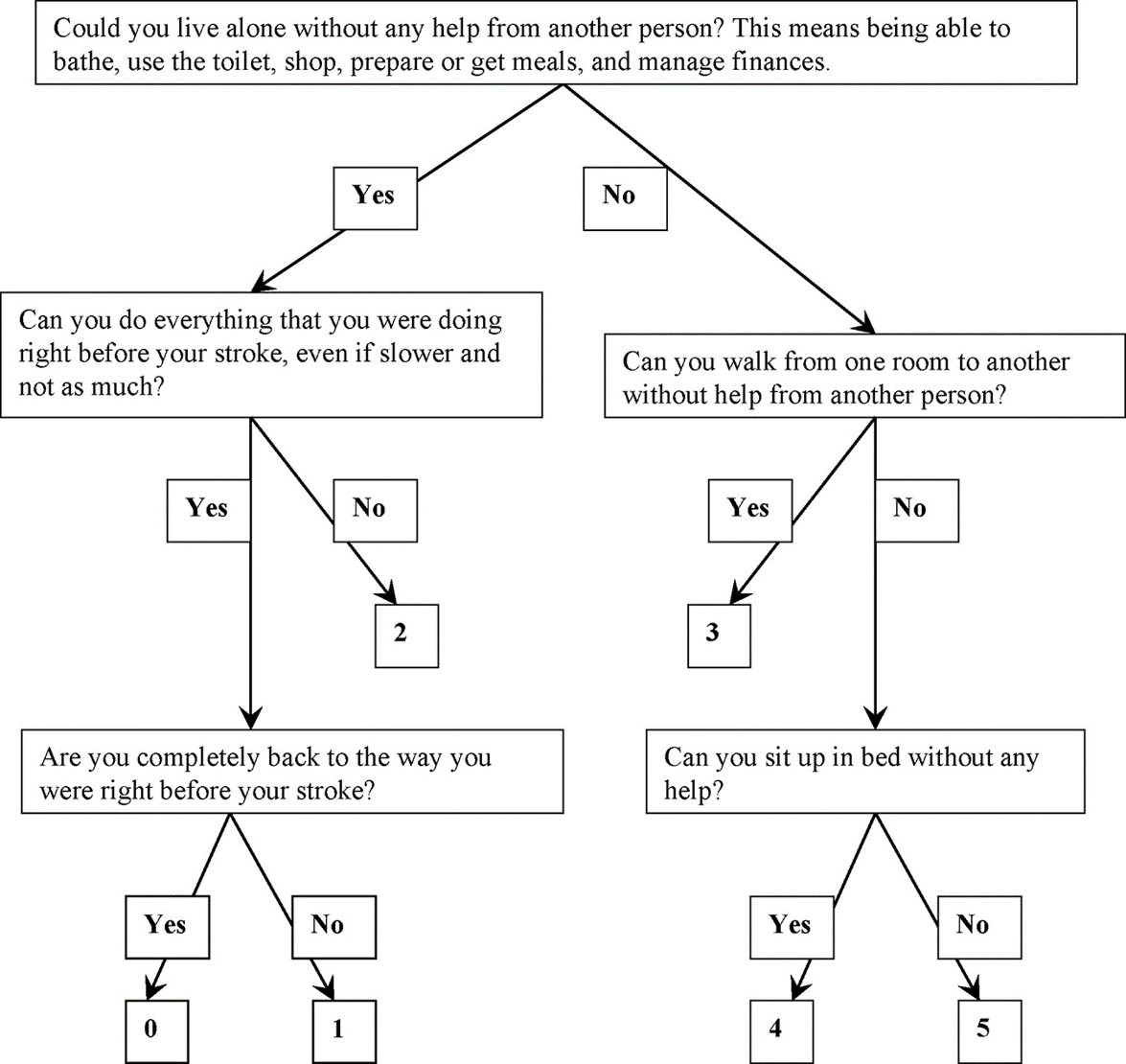


**Appendix 4: Barthel Index for activities of daily living**

To Assesses functional independence in stroke patients

|  | **0 points** | **5 points** | **10 points** | **15 points** |
| --- | --- | --- | --- | --- |
| **Feeding** | Unable | Needs help | Independent | ------------ |
| **Bathing** | Unable | Independent | ------------ | ------------ |
| **Grooming** | Unable | Independent | ------------ | ------------ |
| **Dressing** | Unable | Needs help | Independent | ------------ |
| **Bowel control** | Incontinence (or needs to be given enemas) | Occasional accident | Continent | ------------ |
| **Bladder control** | Incontinence (catheterized and unable to manage alone) | Occasional accident | Continent | ------------ |
| **Toilet use** | Unable | Needs help | Independent | ------------ |
| **Transfers (bed to chair and back)** | Unable | Needs major help (one or two people, physical), can sit | Needs minor help (verbal or physical) | Independent |
| **Mobility on level surfaces** | Immobile or <50 yards | Wheelchair independent, including corners, >50 yards | Walks with help of one person (verbal or physical) >50 yards | Independent (but may use any aid, e.g. stick) >50 yards |
| **Stairs** | Unable | Needs help (verbal, physical, carrying aid) | Independent | ------------ |
| **Total score** |  | | | |

**Interpretation**

- No disability (100)
- Moderate disability (60–95)
- Severe disability (0–55)
